# The structural landscape of Microprocessor Mediated pri-*let-7* miRNAs processing

**DOI:** 10.1101/2024.05.09.593372

**Published:** 2024-05-09

**Authors:** Ankur Garg, Renfu Shang, Todor Cvetanovic, Eric C. Lai, Leemor Joshua-Tor

**Affiliations:** 1 W. M. Keck Structural Biology Laboratory, Cold Spring Harbor Laboratory, One Bungtown Road,Cold Spring Harbor, New York, 11724 USA; 2 Howard Hughes Medical Institute, Cold Spring Harbor laboratory, One Bungtown Road, Cold Spring Harbor, New York, 11724 USA; 3 Developmental Biology Program, Sloan Kettering Institute, 430 East 67th St, ROC-10, New York, NY 10065, USA

## Abstract

miRNA biogenesis is initiated upon cleavage of a primary miRNA (pri-miRNA) hairpin by Microprocessor (MP), composed of the Drosha RNase III enzyme and its partner DGCR8. Multiple pri-miRNA sequence motifs affect MP recognition, fidelity, and efficiency. Here, we performed cryo-EM and biochemical studies of several let-7 family pri-miRNAs in complex with human MP. We show that MP has structural plasticity to accommodate different pri-miRNAs. These also revealed key structural features of the 5’ UG sequence motif, more comprehensively represented as the “fUN” motif. Our analysis explains how the bulged nucleotide in class-II pri-let-7 members alters Drosha cleavage, generating a noncanonical precursor with 1-nt 3’ overhang. Finally, the MP-SRSF3-pri-let-7f1 structure reveals how SRSF3 interacts with the CNNC motif and Drosha’s PAZ-like domain, to promote proper Drosha loading onto the basal hairpin junction. Overall, our work illuminates the mechanisms for flexible recognition, accurate cleavage, and regulated processing of different pri-miRNAs by MP.

## Introduction

Over 60% of the human protein-coding genes are regulated by microRNAs (miRNAs) (Daugaard and Hansen, 2017), underscoring their critical role in many, if not most biological processes. miRNAs are ~ 22 nucleotides (nt) non-coding RNAs that generally repress mRNA expression post-transcriptionally (Shang et al., 2023). miRNAs are processed from hairpin-containing transcripts called primary miRNAs (pri-miRNAs), typically transcribed by RNA polymerase II. In animals, the canonical pri-miRNA stem-loop is cleaved by the Microprocessor (MP), a heterotrimeric nuclear complex containing Drosha, an RNase III endonuclease, and two copies of its essential co-factor, DGCR8. MP cleavage results in a staggered cut in the pri-miRNA to produce a shorter hairpin precursor (pre)-miRNA of ~ 70-80 nt, (Denli et al., 2004; Gregory et al., 2004; Lee et al., 2003) which is exported to the cytoplasm by exportin 5. The cytoplasmic RNase III endonuclease, Dicer, cleaves off the apical loop of the pre-miRNA to generate a ~ 22 base-pair (bp) miRNA duplex. The duplex is loaded into an Argonaute (Ago) protein, where one strand, the guide RNA, remains in the complex forming the mature RNA-induced Silencing Complex (RISC). RISC is then guided to the target RNA, via base-pairing, predominantly through the miRNA seed sequence (nt ~2-8) (Bartel, 2009; Lai, 2002), which ultimately results in target repression.

**Figure-1-:**
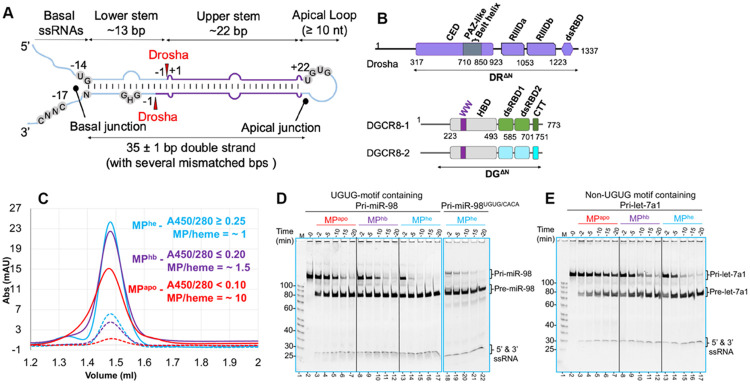
Heme has ubiquitous role in M^2^P^2^. (A) General architecture of a canonical pri-miRNA. The short sequence motifs influencing M^2^P^2^ are highlighted in grey and Drosha cleavage sites are marked with red arrows. (B) Domain architecture of human Drosha (DR) and DGCR8 (DG). The DR^ΔN^ and DG^ΔN^ truncations used in this study are marked. (C) Analytical SEC analysis for different MP heme-variants: MP^he^, MP^hb^ & MP^apo^. The UV280 and UV450 traces are shown in solid and dotted lines, respectively. Average A450/280 ratios and resulting MP/heme molar ratios are shown. (D) The processing assay for pri-miR-98 and (E) pri-let-7a1 by MP^apo^, MP^hb^ and MP^he^. Different RNA species observed are indicated and the 100 nt RNA ladder is marked as “M”.

Apart from its role in initiating miRNA processing, MP acts as a gatekeeper that permits only specific hairpin transcripts, out of an ocean of plausible miRNA-like structures, to enter the biogenesis pathway. The most prominent features are single-stranded flanking regions, a ~ 35 ± 1 bp double-stranded stem often harboring several wobbles, mismatched base pairs, bulges, and a ≥ 10 nt apical loop (Fang and Bartel, 2015; Han et al., 2006; Ma et al., 2013; Zeng and Cullen, 2005; Zeng et al., 2005). In addition, pri-miRNA sequences are enriched for certain short motifs. These motifs affect cleavage efficiency and fidelity of MP-mediated pri-miRNA processing **(M^2^P^2^)** and include a basal UG dinucleotide motif at the −14 nt position in the 5’-strand, a UGUG motif at the 3’ end of 5’ arm (at the base of the apical loop), a CNNC (where N is any nucleotide) motif at the −17 nt position in the 3’-strand and a GHG (where H is not G) mismatch (mGHG) in the 3’-strand at the −3 nt to −5 nt position in the lower stem region (Auyeung et al., 2013; Fang and Bartel, 2015; Nguyen et al., 2015) (Figure 1A). All these motifs are recognized directly by the MP (Fang and Bartel, 2015; Kwon et al., 2016), with the exception of the 3’ CNNC motif, which recruits SRp20/SRSF3 to assist MP in the productive processing of pri-miRNAs (Auyeung et al., 2013; Kim et al., 2018). Nonetheless, pri-miRNAs vary considerably in their structures. This is especially pronounced in the wide variety of sequences, shapes, and sizes of the apical loop, even within a given family of pri-miRNAs, such as the pri-let-7 family (Lee et al., 2016). In addition, a large fraction of miRNAs appears to lack all these motifs. Recent cryo-EM studies provided a glimpse into how the 5’ UG (Jin et al., 2020) and mGHG (Partin et al., 2020) motifs are recognized by the MP. However, the features dictating MP recognition of the large pool of pri-miRNAs lacking these motifs, and the plasticity the MP must have to accommodate the large variation in pri-miRNA structures, remain poorly understood.

The 3’ CNNC motif is the most conserved sequence motif, is present in ~ 60% of human pri-miRNAs, and is specifically bound by a critical splicing factor, SRSF3/SRp20 (Auyeung et al., 2013; Kim et al., 2018; Le et al., 2023). In addition to rescuing the productive M^2^P^2^ of 201 human pri-miRNAs (~11% of the total pri-miRNA pool) in an in-vitro cleavage assay, SRSF3 modulates alternative processing events and suppresses unproductive/abortive processing (nick processing and inverse processing) for hundreds of pri-miRNAs (Kim et al., 2021). Although SRSF3 assists Drosha recruitment at the basal junction for an accurate RNA cleavage (Kim et al., 2018), a mechanistic view of SRSF3’s effect on pri-miRNA processing remains unknown.

One of the first miRNA families discovered, the let-7 miRNAs, contains 12 functionally conserved members (Bussing et al., 2008). MP processing categorizes these pre-let-7s into two distinct classes: three pre-let-7s belong to class-I with the canonical 2-nt 3’overhang, and nine belong to class-II with just a non-canonical 1-nt overhang at the 3’ end (Lee et al., 2016). Class-II pre-let-7s are specifically mono-uridylated by a Terminal Uridylyl transferase-(TUT)4/7 (Faehnle et al., 2017; Heo et al., 2012; Thornton et al., 2014) to convert them to a suitable substrate for Dicer, which prefers a 2-nt 3’ overhang substrates, ensuring their efficient processing into the mature miRNAs (Park et al., 2011).

A bulged nucleotide at the 5’ cleavage site in class-II pri-let-7 members is thought to be responsible for their non-canonical M^2^P^2^ (Heo et al., 2012). However, the molecular basis for this difference in processing is still unclear.

In this study, we took a structural and biochemical approach to understand how the MP handles the wide range of pri-miRNA substrates, including class-I and class-II pri-let-7 family miRNAs, expand our understanding of MP motif recognition, and uncover how SRSF3 modulates the M^2^P^2^ for CNNC containing pri-miRNAs.

## Results

### Heme facilitates M^2^P^2^

Heme plays a crucial role in M^2^P^2^ (Weitz et al., 2014), by promoting HBD dimerization (Faller et al., 2007) and engagement of the apical UGUG motif. However, the specific involvement of heme is not fully understood. We studied the effect of heme binding in the MP complex on pri-miRNA processing, by expressing and purifying the heme-free MP complex (MP^apo^) away from endogenous heme-bound MP (MP^hb^) (Figure S1A). Next, we expressed the MP complex by supplementing the expression media with 0.75 mM 5-aminolevulinic acid hydrochloride (5-ALA), an intermediate in the heme biosynthesis pathway. Purified MP complex elutes as a yellow-colored heme-enriched (MP^he^) single species (Figure S1B). The average UV 450/280 absorbance (A450/280) ratio for purified MP^he^, MP^hb^ and MP^apo^ was ≥ 0.25, ≤ 0.20 and < 0.10, respectively (Figure 1C). The calculated “MP/heme” molar ratio for MP^he^, MP^hb^ and MP^apo^ proteins were ~ 1, ~ 1.5 and ~ 10, respectively, indicating differential heme occupancies in these forms of purified MP complexes.

We subjected these to an *in-vitro* time-course cleavage assay using pri-miR-98, a UGUG-motif containing pri-let-7 pri-miRNA, as a substrate. We observed that the MP^he^ processed of pri-miR-98 into pre-miR-98 is significantly faster than, either with MP^hb^ or MP^apo^, indicating that MP activity on the UGUG-motif containing pri-miR-98 substrate is heme-dependent (Figure 1D). This underscores the importance of utilizing properly reconstituted MP complex for functional characterization, especially recombinantly expressed protein. Mutation of the UGUG motif in pri-miR-98 to CACA, results in slower processing by MP^he^, suggesting that the UGUG sequence motif indeed plays a role in M^2^P^2^ of pri-miR-98 (Figure 1D **compare lane 13-17 to 18-22**). Interestingly, similar RNA processing differences were observed for pri-let-7a1 (a non-UGUG motif containing pri-miRNA), demonstrating the role for heme in non-UGUG motif containing pri-miRNA processing as well (Figure 1E).

Incubating MP^he^ with different pri-let-7 family members exhibited significant variability in rates of RNA processing. Pri-miR-98 and pri-let-7g are cleaved into their respective pre-miRNAs much faster than pri-let-7a1. On the other hand, pri-let-7c substrate is processed in an inverse orientation very quickly, with almost no canonical pre-let-7c product produced (Figure S1C). Interestingly, MP^apo^ exhibits much slower processing rates for all tested pri-let-7 miRNAs. The control pri-miR-16-1 RNA (a non-UGUG motif containing miRNA), showed a significantly slower processing rate when incubated with MP^apo^ as compared to MP^he^ as well (Figure S1D). Overall, these results strongly suggest that heme plays a crucial role in M^2^P^2^ of not only the UGUG-motif containing pri-miRs, but the non-UGUG motif containing pri-miRs as well, and is likely linked to the integrity of the trimeric MP complex.

### The pri-let-7s-MP^he^ complex reveals new features (Class-II let-7s)

**Figure-2-:**
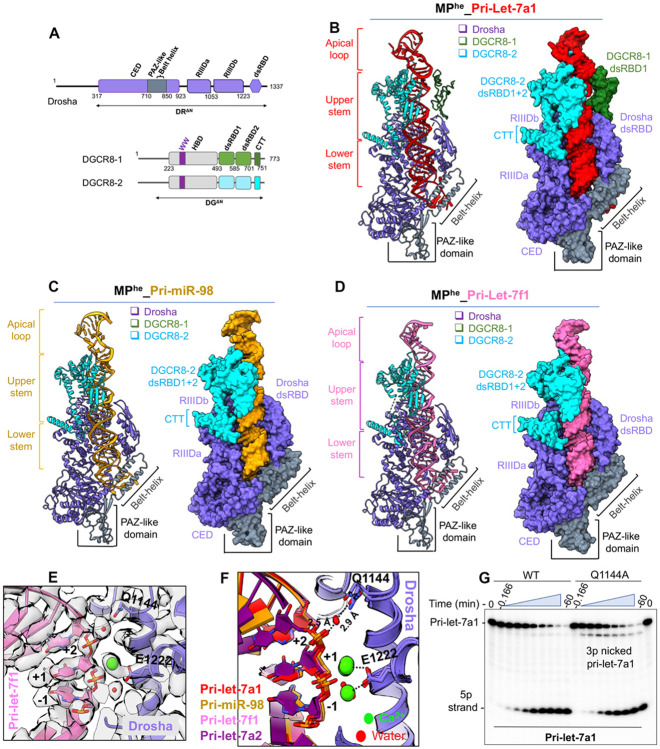
Cryo-EM structure of MP^he^ & class-II pri-let-7s (A) Domain architecture of human Drosha (DR) and DGCR8 (DG). The DR^ΔN^ and DG^ΔN^ truncation used for cryo-EM analysis. (B) Cartoon and space fill representation of the cryo-EM structure of MP^he^-pri-let-7a1, (C) MP^he^-pri-miR-98 and (D) MP^he^-pri-let-7f1 complex. The complete pri-miRNA stem and the apical loop is visible in the structure with the UGUG motif exposed. (E) The cryo-EM density in the 5’ catalytic site of MP^he^-pri-let-7f1 map showing the well-coordinated water molecules (red) and Ca^2+^ ion (green). (F) Multiple pri-let-7s structures showing the conserved water stabilizing the 5p +2 geometry via Q1144. (G) pri-let-7a1 processing assay with MP^he^-Q1144A mutant showing accumulation of 3’ nicked RNA products compared to MP^he^.

To understand how the MP accommodates the large variation in pri-let-7 pri-miRNAs we determined cryogenic electron microscopy (cryo-EM) structures of MP^he^ in complex with three pri-let-7s in their pre-catalytic state. For these studies, we used an N-terminal truncation of human Drosha isoform 4, DR^ΔN^ (amino acids 317-1337), and an N-terminal truncation of human DGCR8 DG^ΔN^ (amino acids 175-751) (Figure 2A). We will be using the more common Drosha isoform 1 numbering for consistency. The overall resolutions for the cryo-EM maps were 3.2 Å, 3.3 Å and 2.9 Å for pri-miR-98, pri-let-7a1 and pri-let-7f1 bound to MP^he^, respectively, as estimated from their GSFSC curves (Figure S2A, S2D, S2G). Although local resolution varies for these structures, Drosha and most of the pri-miRNAs were observed at high resolution for all three, while DGCR8 and the apical loop of the pri-miRNA were at lower resolution (Figure S2B, S2E, S2H).

Overall, our MP^he^-pri-let-7 structures display the familiar canonical heterotrimeric arrangement of the Drosha-(DGCR8)_2_ complex (Figure 2A-D) (Jin et al., 2020; Kwon et al., 2016; Partin et al., 2020) trapped in a pre-catalytic state. The heterotrimer is stabilized by the interaction of Drosha RIIIDa and RIIIDb with the CTT peptides from DGCR8-2 and DGCR8-1, respectively. Both dsRBDs from DGCR8-2 were visible and could be built in all three structures (Figure 2B-D), whereas only the MP^he^-pri-let-7a1 cryo-EM map had good enough density to build DGCR8-1’s dsRBD1 (Figure 2B). However, DGCR8-1 dsRBD2 could not be built in any of the structures due to the lack of defined cryo-EM density, and is likely flexible due to the absence of any stabilizing interactions. All the cryo-EM maps show a significant chunk of density above the DGCR8-dsRBDs, which corresponds to the RNA apical loop and the DGCR8 HBD dimer (Figure S3A-B). Cryo-EM map denoising allowed us to visualize partial secondary structure features in this density, but due to lack of any guiding structure for this region, we could not build the complete HBD into the map. In addition, AlphaFold (Jumper et al., 2021) prediction models do not match the observed density (Figure S3C-D). Nonetheless, the crystal structure of the dimerization subdomain, comprised of the WW motif (PDBid 3LE4) (Senturia et al., 2010) could be confidently docked into the map away from the RNA. The WW motif presents the interaction surface for heme and includes two cysteines that act as ligands to the heme iron (Faller et al., 2007; Senturia et al., 2012), though the heme group itself was not resolved in any of our structures nor in the crystal structure. Interestingly, this subdomain has no direct contact with the RNA (Figure S3A-B).

In all three structures, the pri-let-7 stem adopts a near A-form helical conformation over ~ 3.2 helical turns, covering ~ 35 base pairs. As observed in previous studies, the dsRNA-ssRNA junction (called the basal junction) is clamped between an α-helical hairpin (the ‘belt’) emanating from the PAZ-like domain, and the wedge loop (amino acids 930-952) from Drosha (Jin et al., 2020; Partin et al., 2020). Of this, two turns of the RNA double-helix (~ 22 bp) are docked onto the central groove formed between the two RNase III domains (RIIID) and dsRBD of Drosha, while ~ 1.2 turns (~ 13 bp) are engulfed by the DGCR8 dsRBDs (Figure 2B-D). Thus, the RNA in the pre-catalytic state is stabilized via interactions with all three proteins of the complex all along the RNA backbone.

The 5’ and 3’ RNA cleavage sites for the different pri-let-7s are positioned at the catalytic sites of RIIIDb and RIIIDa, respectively. A Ca^2+^ ion, used in place of the Mg^2+^ ion to prevent catalysis, is accommodated in either one or both RIIIDs in the different structures. However, the observed density for the Ca^2+^ ion in RIIIDb is consistently stronger than at the RIIIDa site, likely due to the differential binding of Ca^2+^ ions in the two RIIIDs, as was observed previously (Partin et al., 2020). Additionally, our cryo-EM maps revealed a conserved water molecule stabilizing the 5p +2 nt phosphate via Gln1144 from RIIIDb, anchoring the RNA backbone and rigidifying the downstream 5’ cleavage site (Figure 2E). This water-mediated interaction is observed in all our structures (Figure 2F and Figure S3E-H). Mutation of Gln1144 to an alanine affects the 5p strand cleavage for pri-let-7a1, generating significantly more 3’ nicked RNA products (Figure 2G). In the 5’ cleavage site, Glu1222 was known to coordinate the catalytic Mg^2+^ ion (Kwon et al., 2016; Nguyen et al., 2015) and our study shows that Gln1144-mediated 5p +2 phosphate stabilization is also important for efficient pri-miRNA cleavage.

An RNA stem of ~ 35 bp in length is a hallmark of canonical pri-miRNAs (Fang and Bartel, 2015, 2020; Han et al., 2006). The cryo-EM structures reported here now show the complete 35 to 36 bp RNA stem (Figure 2B-D). Analysis of the different MP-pri-let-7s structures confirms that the pri-let-7 stem can easily accommodate many non-Watson-Crick (WC) bps at different positions, without major perturbations in the RNA helix (Fang and Bartel, 2015). The UGUG motif (in pri-miR-98 and pri-let-7f1) and the Lin28-interacting GGAG/GAAG motif (in pri-let-7a1) (Mayr et al., 2012; Nam et al., 2011), both in the apical loop, could be clearly observed. Interestingly, the UGUG motif is solvent exposed, while the GGAG/GAAG motif abuts the unmodelled HBD density. Cryo-EM maps exhibited helical density atop the RNA stem region (Figure S3A-B) and aided by the crystal structure of the Lin28-pre-let-7 complex (PDBid 5UDZ) (Wang et al., 2017), we were able to model this density as the part of the apical loop in different pri-let-7s. The apical loop in each of the three pri-let-7s adopts a hairpin structure with ~ ½ turn of dshelical geometry, dominated by G-C base-pairing. However, the nucleotides corresponding to the unpaired loop region could be built with confidence only for the pri-let-7a1 structure (Figure 2B). Interestingly, this part of the RNA helix along with the unpaired loop nucleotides in the apical loop is exposed in all three MP^he^-bound structures, and could serve as a point of contact for other RNA-binding proteins (RBPs) that might modulate M^2^P^2^ activity. Overall, we were able to view the almost complete pri-let-7s bound to MP^he^ in a pre-catalytic state in these cryoEM structures.

### RNA drives MP domain repositioning for pri-let-7 cleavage

MP selectively processes pri-let-7 miRNAs into class-I or class-II pre-let-7s, leaving either a canonical 2-nt or just 1-nt 3’ overhang, respectively (Heo et al., 2012). To understand the basis for this discrepancy, we determined a 2.9 Å Cryo-EM structure of MP^he^ with pri-let-7a2, a class-I let-7, (Figure S4A-B) in the pre-catalytic state, and compared it with pri-let-7a1, pri-let-7f1 and pri-miR-98 bound MP^he^ structures described above, all belonging to class-II let-7.

**Figure-3-:**
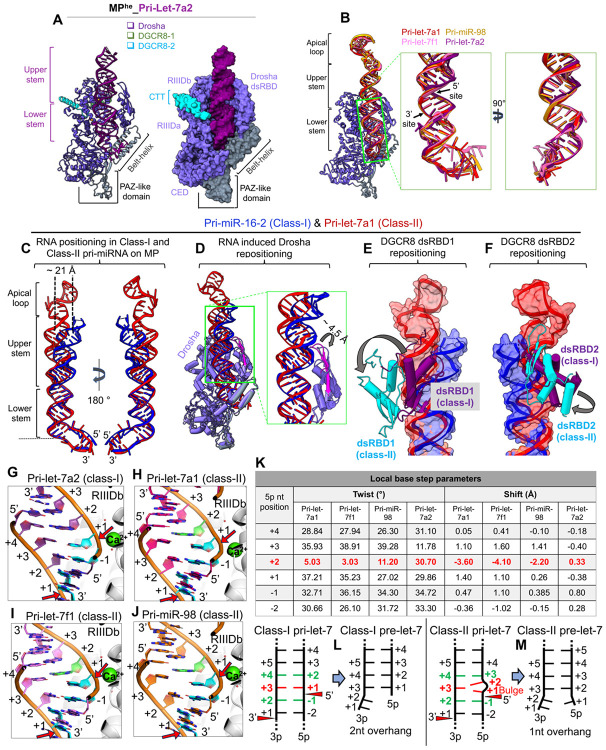
RNA-driven structural rearrangements in MP in the two pri-let-7 classes. (A) Cartoon and space-filling representation of the cryo-EM structure of the MP^he^-pri-let-7a2 complex. (B) Drosha superimposition showing the conserved positioning of the RNA lower stem in different pri-let-7s in their pre-catalytic state. (C) Superimposition of the class-I (pri-miR-16-2 in blue) and class-II (pri-let-7a1 in red-orange) pri-miRNA bound on MP^he^ in pre-catalytic state. The bending of class-II RNA upper stem displays a ~21 Å lateral shift in the bases of their apical loops. The MP^he^ has been removed for clarity. (D) Drosha superimposition among the class-I (pri-miR-16-2 in blue) and class-II (pri-let-7a1 in red-orange) pri-miRNA bound MP^he^ structures. Drosha dsRBD β-sheet moves to adjust for the local helical bending in class-II pri-let-7. The repositioning of DGCR8 dsRBD1 (E) and dsRBD2 (F) bound to class-I (pri-miR-16-2 in blue) and class-II (pri-let-7a1 in red-orange) pri-miRNA upper stem. dsRBDs repositioning are shown with the arrows. Base-pairing patterns at the 5’ cleavage site in (G) class-I pri-let-7a2, and class-II (H) pri-let-7a1 (I) pri-let-7f1 and (J) pri-miR-98. The 5p +1nt (cyan) in pri-let-7a2 is paired with 3p +3nt, while it remains unpaired/bulged in class-II structures. Cleavage sites are highlighted with red arrows. (K) The local base twist and shift parameters for the 5p nucleotides around the 5’ cleavage site. The 5p +2 shows distorted geometry (highlighted in red) accommodating for the bulged 5p +1 nt. A schematic of the base-pairing patterns observed in class-I pre-let-7 (L) and class-II pri-let-7s (M) when bound to MP in their pre-catalytic state. Drosha cleavage sites are shown with red arrows. The bulged 5p +1 nt in class-II pri-let-7s stays unpaired and changes the upstream base-pairing register, allowing MP to generate pre-let-7 species with a 1nt 3’ overhang.

Class-II let-7 pri-miRNAs are characterized by the presence of a bulged nucleotide immediately following the 5’ cleavage site. The MP^he^-pri-let-7a2 cryo-EM structure shows Drosha to be positioned on the RNA basal junction, with its dsRBD interacting with the RNA upper stem region (Figure 3A). Notably, both DGCR8-CTTs interact with Drosha, though in this structure cryo-EM density corresponding to the DGCR8-HBD-dsRBDs and a portion of pri-let-7a2’s upper stem and apical loop was not well resolved (Figure S4C). Drosha is positioned almost identically in all four structures on the RNA basal junction, with an almost identical lower stem RNA geometry in all the pri-let-7s structures described here (Figure 3B), as well as in the structure of the complex with pri-miR-16-2 (Partin et al., 2020). However, the RNA upper stem in class-I pri-let-7a2 is bent compared to the class-II pri-let-7 miRNAs (Figure S4D). Similarly, the class-I type pri-miR-16-2 RNA (Partin et al., 2020) also exhibits a bend of the upper stem relative to the Drosha-lower stem RNA complex (Figure 3C), Notably, the kink in the helix starts right after the 5’ cleavage site, which results in a lateral displacement of up to 10 Å and 21 Å of the 3p and 5p nucleotides at the +20^th^ base-pair position in the upper stem, respectively (Figure 3C).

Bending of the RNA upper stem is complemented by structural rearrangements in both Drosha and DGCR8. The core of Drosha, including the CED and the RIIIDs, shows no significant changes among the different pri-let-7 bound structures, apart from the Drosha dsRBD’s β-hairpin (β1-β2) that opens ~4.5 Å from the rest of the domain to accommodate the movement of the RNA upper stem in class-I pri-miRNA (pri-miR-16-2 in the figure) (Figure 3D), retaining similar contacts with the RNA. All three observed dsRBDs spatially reposition themselves with the RNA upper stem in class-II pri-let-7a1 compared to class-I pri-miR-16-2 (Figure 3E-F) such that all the RNA specific interactions with MP dsRBDs are conserved in both class-I and class-II pri-miRs. This indicates that the MP undergoes an RNA-induced conformational rearrangement, accommodating the changes in RNA structure. Taken together, the different cryo-EM structures reveal the plasticity of MP’s dsRBDs to recognize and incorporate different pri-let-7 miRNAs, driven by rearrangements in the RNA upper stem.

### Generation of class-I vs. class-II pre-miRNAs

The class-II bulged nucleotide at the +1 position of the 5’ strand was long suspected as the culprit in the difference in the 3’-overhang in the MP cleavage products between class-I and class-II pre-miRNAs (Heo et al., 2012). To understand how MP generates the two different 3’ overhangs, we compared the two classes of pri-let-7 RNAs around the cleavage sites. We found that in class-I pri-let-7a2 the 3p +2, +3 and +4 nts pair up with the 5p −1, +1, and +2 nts, respectively (Figure 3G). For class-II pri-let-7 structures the 3p +2 nt also pairs with the 5p −1 nt, but with the bulge at the 5p +1 position unpaired, 3p +3 now pairs with the 5p +2 nt, and the nts that follow are all shifted by one (Figure 3H-J). The 5p +2 nt appears to absorb most of the distortion from the presence of the bulge immediately preceding it. It exhibits a very low base twist and significantly high base shift (Figure 3K), with its backbone bulging outward from the RNA helix (Figure S4E). Its base pair with 3p +3 exhibits significant base-pair shearing and higher base-pair step-rise (Figure S4F-G). This geometry allows the RNA duplex to accommodate the bulge. The bulge at 5p +1 stacks on top of the 5p −1 base and is on a plane with the 3p +3 base without base pairing with it (Figure 3H-J). The consequence of these acrobatics is that it “straightens out” the overall trajectory of the duplex above the cleavage site compared to class-I pri-miRNAs (**see above and** Figure 3C). In addition, this positions the 5p +1 bulge nucleotide above the catalytic ion, resulting in the inclusion of an extra nucleotide in 5p strand, leaving only a single nt as the overhang thus generating class-II pre-let-7s (Figure 3L-M).

### The UG or fUN motif

**Figure-4-:**
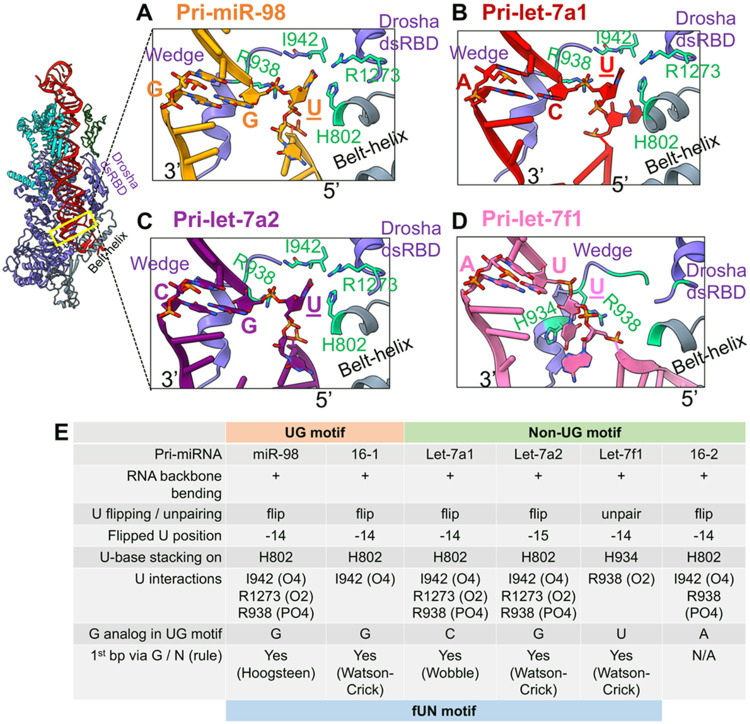
The 5’ UG vs fUN motif? A zoomed-in view of the interactions observed in the UG (or fUN) motif in (A) pri-miR-98, (B) pri-let-7a1, (C) pri-let-7a2 and (D) pri-let-7f1 with residues (green sticks) from, Drosha’s wedge, dsRBD, and the belt-helix. The U flips out (underscored) while N establishes the 1^st^ base-pairs in the RNA lower stem. (E) Conserved structural features characteristic of this motif.

The UG motif is prevalent in ~25% of human pri-miRNAs, and is located at the 5p −14nt position, at the basal junction (Auyeung et al., 2013). The UG motif is tightly enclosed by Drosha’s belt, the wedge loop, and its dsRBD. The cryoEM map of this region is at high resolution in all our structures. Consequently, we could analyze the different pri-let-7s with high confidence, which revealed unique structural features associated with this region in human pri-miRNAs in complex with MP. The U of this motif in the MP^he^-pri-miR-98 structure is flipped out with its base stacking onto His802 from one of the helices of the Drosha belt. The identity of the base is recognized through hydrogen bonding interactions between O2(U) and O4(U) and Arg1273 (from Drosha dsRBD) and Ile942 main chain amide (from Drosha wedge), respectively (Figure 4A). Its phosphate interacts with Arg938 (from the wedge loop). The following G in pri-miR-98, at position −13, pairs with a G on the 3’ strand via its Hoogsteen edge establishing the first base pair in the lower stem (Figure 4A). The pri-miR-16-1 structure (PDBid 6LXD) is the only other UG motif containing pri-miRNA structure reported, which exhibits similar interactions for this motif except that −13 G pairs with a C on the 3p strand through canonical Watson-Crick hydrogen bonding (Jin et al., 2020) (Figure S4H).

Many pri-miRNAs lack the UG motif *per se*; however, in some cases, there is a U at position −14 on the 5’ strand nonetheless. Three of our structures, the MP^he^ complexes of pri-let-7a1, pri-let-7a2 and pri-let-7f1 do not contain a UG motif, but all have an unpaired U at the −14^th^ position, or −15^th^ position (in pri-let-7a2), and in all but one (pri-let-7f1), this U is flipped out (Figure 4A-D). Moreover, the U-specific interactions are conserved in most of these MP-pri-miRNA structures, with the exception of the complex with pri-let-7f1. In that case, the U base stacks onto His934 (from the Drosha wedge loop); it is the O2 atom that interacts with Arg938 (from the wedge loop) rather than its phosphate, as observed in the other structures. Similar to the pri-miRNAs with a UG motif, the following nucleotide in all these structures pairs with a nucleotide from the 3’ arm to form the 1^st^ base-pair of the lower stem, either through canonical Watson-Crick (Figure 4C-D) or via non-canonical base pairing (Figure 4A-B). In the structure with pri-miR-16-2 RNA (PDBid 6V5B) the U at position −14 is flipped out as well, but the following nucleotide stays unpaired (Figure S4I).

Overall, our structural analysis shows that whether the pri-miRNA has a strict UG motif or not, the U at position −14 is flipped or is at least unpaired and is sensed by several amino acids from Drosha, while the neighboring nucleotide at the −13 position, forms the first base pair in the RNA lower stem, whether it is a G or not (Figure 4E), though a strong G enrichment at the −13 position was reported in a previous study (Auyeung et al., 2013). Our analysis reveals that there are strong structural features associated with U at the −14 position in 5p strand of pri-miRNAs. We, therefore, propose to revise the ‘UG sequence motif designation to a ‘flipped U with paired N’ **(fUN)** structural motif positioned predominantly at the −14 position of the 5p strand. The fUN motif designation would result in a more accurate classification of the pri-miRNA pool.

### SRSF3-assisted M^2^P^2^ via the 3’ CNNC motif

**Figure-5-:**
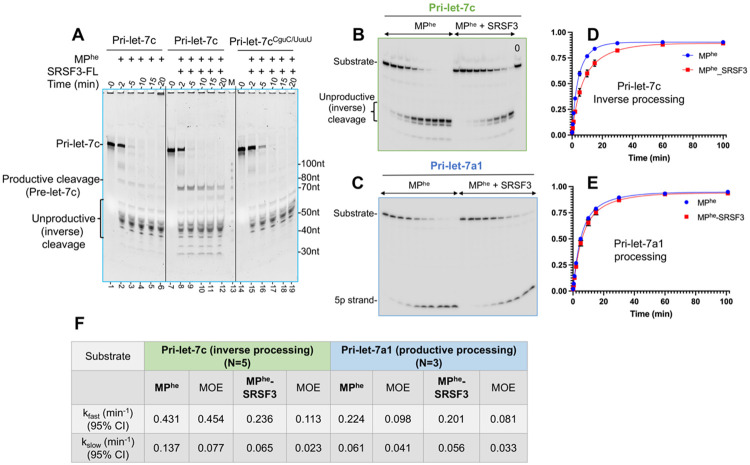
SRSF3 assists Drosha loading on the basal junction via the 3’ CNNC motif. (A) *In-vitro* processing of pri-let-7c and pri-let-7c^CNNC/UUUU^ by MP^he^ and SRSF3. Significantly more pre-let-7c is produced when SRSF3 is added to the pri-let-7c reaction. The RNA substrate, productive cleavage products and unproductive cleavage products are indicated. “M” denotes a 100nt RNA ladder. A representative urea-PAGE gel of 5’ ^32^P-labelled RNA processing assay for (B) CNNC motif-containing pri-let-7c and (C) non-CNNC motif-containing pri-let-7a1. (D) The fit for the quantified unproductive cleavage products for pri-let-7c and (E) Fit for productive cleavage products for pri-let-7a1. (F) Calculated observed rate constants for the pri-let-7c inverse processing and pri-let-7a1 processing. N values for each substrate is also marked. MOE is margin of error.

As mentioned, SRSF3 impacts the processing of hundreds of CNNC motif-containing pri-miRNAs, including those of the pri-let-7 family. To determine the mechanism involved, we recombinantly expressed and purified human SRSF3 (Figure S5A), and included it in our time course processing assay. We chose the CNNC motif-containing pri-let-7c (CGUC) as a substrate because, *in vitro,* MP^he^ processes it primarily in an inverse orientation, resulting in unproductive cleavage, and produces only a minimal amount of the canonical pre-let-7c product (productive cleavage) (Figure 5A **lane 1-6 and** Figure S1C **lane 8-13)**. This behavior makes pri-let-7c very useful for comparative analysis against SRSF3 variants, as any improvement in pre-let-7c yield could be reliably observed and quantified.

Adding SRSF3-FL to the RNA cleavage reaction significantly improved productive cleavage of pri-let-7c (Figure 5A**: compare lanes 8-12 to lanes 2-6 and** Figure S5B). An increase in the canonical pre-let-7c product underscores SRSF3’s role in positioning Drosha at the RNA basal junction in a stable manner, as observed for pri-miR-30a (Kim et al., 2018). Using a truncation of SRSF3 encompassing the RRM alone, SRSF3-RRM (amino acids 1-84), shows a much-reduced level of the pre-let-7c product, suggesting that the SR-rich region of SRSF3 might be involved in the Drosha assist (Figure S5C **lane9-13)**. Furthermore, a CNNC mutation to UUUU resulted in loss of SRSF3-mediated enhancement of pri-let-7c^CNNC/UUUU^ productive cleavage (Figure 5A **lane14-19),** confirming the requirement for the CNNC motif in the Drosha assist. As expected, for the CNNC-deficient pri-let-7a1, no effect was observed upon addition of SRSF3 in a 30-min time course assay (Figure S5C **lane14-25)**. Additionally, for another CNNC-containing RNA, pri-let-7g, the effects on M^2^P^2^ are better observed in the unproductive cleavage products, which are substantially reduced when incubated with SRSF3 (Figure S5D**: compare lane 2-6 with lane 8-12)**. Moreover, CNNC mutation within pri-let-7g increased levels of inverse cleavage products (Figure S5D **lane 14-19**) underscoring the motif’s importance.

We then quantitatively characterized the effect of SRSF3 on M^2^P^2^ of 1) the CNNC-containing pri-let-7c, and 2) the CNNC-deficient pri-let-7a1. Since M^2^P^2^ of pri-let-7c results predominantly in unproductive cleavage, we compared the unproductive cleavage products (inverse processing rate) for pri-let-7c with and without SRSF3 (Figure 5B). The pri-let-7c processing traces were fitted with a biphasic decay isotherm, providing the observed rate constant (k_obs_) for individual steps (k_fast_ and k_slow_). Inverse cleavage products for this reaction result in k_fast_ and k_slow_ of 0.431 ± 0.45 min^−1^ and 0.137 ± 0.07 min^−1^, respectively, which slows down to almost half (k_fast_ and k_slow_ of 0.236 ± 0.11 min^−1^ and 0.065 ± 0.02 min^−1^ respectively) when SRSF3 is added to the reaction (Figure 5D, 5F).

Notably, for the CNNC-deficient pri-let-7a1, SRSF3-FL does not alter the rate for either substrate disappearance or product accumulation (Figure 5C, E). The measured k_fast_ and k_slow_ for pri-let-7a1 catalysis varies from 0.224 ± 0.09 min^−1^ and 0.061 ± 0.04 min^−1^ for MP^he^ alone, to 0.201 ± 0.08 min^−1^ and 0.056 ± 0.03 min^−1^ in the presence of SRSF3-FL (Figure 5F), confirming the lack of SRSF3 involvement in non CNNC pri-miRNAs processing. Overall, our results suggest that SRSF3 assists in the M^2^P^2^ via the 3p CNNC motif in pri-let-7s, which slows their inverse processing, thus improving productive cleavage.

### The cryoEM structure of the MP^he^-pri-let-7f1-SRSF3 complex

To understand how SRSF3 ensures M^2^P^2^ fidelity, we determined a 3.1 Å cryo-EM structure of the quaternary complex containing MP^he^-pri-let-7f1 with SRSF3-FL assembled in a pre-catalytic state (Figure S6A, E). The cryo-EM map revealed unambiguous density not only for the MP and the majority of the RNA but also for SRSF3 and, importantly, for the region of the RNA encompassing the CNNC motif (Figure S6B-D). The MP^he^-pri-let-7f1 structure was confidently placed into the cryo-EM map, revealing extra density near Drosha’s PAZ-like domain and the belt. This density corresponds to SRSF3 and the 3p strand of the pri-miRNA extending beyond the CNNC motif. We used the NMR structure of the SRSF3-RRM domain (PDBid 2I2Y) (Hargous et al., 2006) for initial placement into the extra density. We then identified a short linker peptide (thumb peptide) from the RS domain of SRSF3 and the extended 3p RNA strand, which were manually built into the map.

**Figure-6-:**
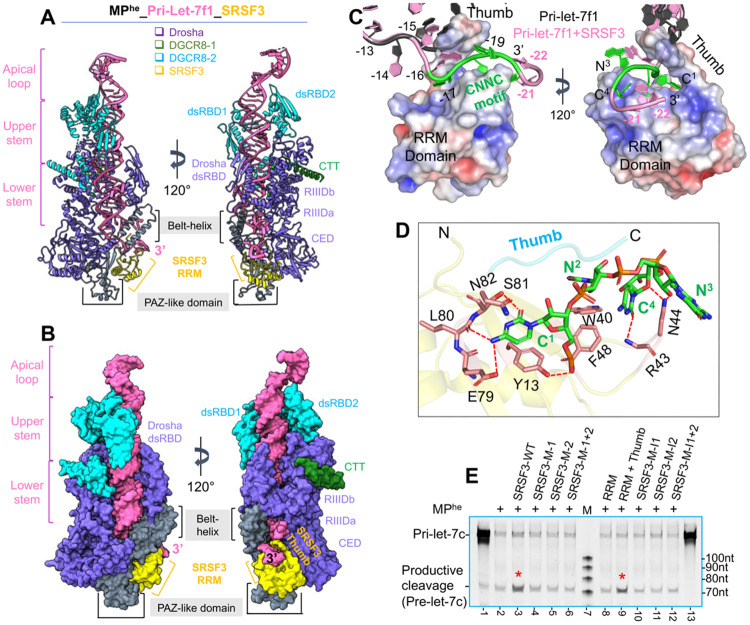
The MP^he^-pri-let-7f1-SRSF3 quaternary complex. (A) Two views of the cryo-EM structure of MP^he^-pri-let-7f1-SRSF3 in the precatalytic state are shown in cartoon and (B) surface representation. The almost complete pri-let-7f1 in resolved (pink). The extended 3p RNA strand with CNNC motif bound by SRSF3 RRM domain (yellow), which is docked onto Drosha’s PAZ-like domain (grey). The thumb peptide is clearly visible, and interleaved between the two RNA strands. (C) The pri-let-7f1 3p ssRNA region in the MP^he^ (dark grey) and MP^he^-SRSF3 structures (pink) shown in two orientations. The 3p strand in the MP^he^ structure (dark grey) would clash with the SRSF3 thumb peptide. In the MP^he^-SRSF3 bound structure, the CNNC motif (green) passes through the electrostatically charged channel formed by the SRSF3 RRM domain and thumb. Mapping of the electrostatics on the SRSF3 surface is shown (−5 (red) to +5 kT (blue)). (D) Molecular interactions between the CNNC motif (green sticks) and SRSF3. C^1^ establishes several H-bonds, and N^2^ and C^4^ stack against residues in the RRM domain (beige colored sticks). Direct H-bonding interactions are shown as red dotted lines. (E) *In-vitro* M^2^P^2^ of pri-let-7c with different SRSF3 mutations/truncations. Only the RRM-thumb restores pre-let-7c (marked) levels to SRSF3-WT level. SRSF3-M-1, M-2 and M-1+2 indicate SRSF3 with mutations in C^1^, C^4^ or C^1^+C^4^ nucleotide-stabilizing residues in the CNNC motif, respectively. RRM and RRM thumb denotes SRSF3^1-84^ and SRSF3^1-90^ truncations. SRSF3-M-I1, M-I2 and M-I1+2 are SRSF3 with mutations in the Drosha interface 1, 2 or 1+2 respectively. “M” denotes a 100nt RNA ladder.

The MP^he^-pri-let-7f1-SRSF3 structure shows Drosha and DGCR8 interacting with the basal junction and upper stem, respectively, as in the structures described above. Both dsRBDs and the CTT of DGCR8-1 were observed in the quaternary structure, while only the CTT for DGCR8-2 was observed (Figure 6A-B). Similar to other MP^he^-pri-let-7 structures, low-resolution density for the HBD was present in the cryoEM map (Figure S6C-E) but could not be modeled. The pri-let-7f1 RNA adopts a helical geometry over the length of the stem (32 bp), with both RNA strands positioned close to the Ca^2+^ ions in the RIIID domains in the pre-catalytic state. As expected, the RNA stem in the quaternary complex is in a typical class-II pri-let-7 conformation **(see section 3, and** Figure 3C, S4D), with the dsRBDs spatially adapted to the position of the RNA **(see** Figure 3D-F).

In this structure, the 3p strand is resolved to position 22, which includes the complete CNNC motif, while only 17 were well-ordered in the MP^he^-pri-let-7f1 ternary structure. Though SRSF3-FL was used for the cryo-EM sample preparation, we could clearly position the RRM domain and a portion of the RS domain (amino acids 1-87) (Figure 6B), but density for the remainder of the RS domain (amino acid 88-164) was not observed, suggesting that it might not be involved in direct interactions with the MP-pri-miRNA complex in the pre-catalytic state. Nevertheless, the local resolution of SRSF3 and the RNA in that region is 3.0 – 4.0 Å (Figure S6E). The MP^he^-pri-let-7f1 geometry is largely unchanged upon SRSF3 binding, and all the MP^he^ RNA interactions remain intact. Notably, SRSF3 grabs onto the CNNC motif in the extended 3p RNA strand, like a hand gripping a rope, and nestles onto the preformed Drosha PAZ-like domain surface, burying ~703 Å^2^ surface area to form a new protein-protein interface (Figure 6A-B). The Drosha PAZ-like domain exhibits only minor structural rearrangements upon SRSF3 binding with an RMSD of 0.26 Å over 70 Cα atoms.

### CNNC motif recognition

The SRSF3 RRM domain adopts a canonical βαββαβ fold (Maris et al., 2005) (Figure S7A) with several surface-exposed hydrophobic residues in its β-sheet (Figure S7C). Interestingly, the SRSF3 linker peptide (amino acids 84-87) folds on top of the β-sheet to create a positively charged narrow channel suitable for RNA binding. SRSF3 clamps the RNA CNNC motif (also denoted as 3p −17 C^1^N^2^N^3^C^4^ motif in the text below) between its “thumb” (the linker peptide) and ‘fingers’ (the RRM β-sheet) (Figure 6C and Figure S7B). The SRSF3 thumb peptide is inserted between the two RNA strands, gripping the 3p strand, ensuring that the 3p −15, −16, −17 and −18 nucleotides do not base pair with the 5p strand, as has been observed in some of the ternary structures described above (pri-let-7f1, pri-miR-98) and elsewhere (Jin et al., 2020). Arg86 from the thumb peptide is inserted between the −15 and −16 bases, and distorts the RNA backbone, positioning the −17 C^1^ base in the RNA binding channel on SRSF3 (Figure 6C). The C^1^ base is stacked between Asn82 and Tyr13 (from β1) and establishes multiple H-bonds with Glu79, Ser81 side chains and the Leu80 backbone oxygen. Also, the C^1^ phosphate is stabilized by a H-bond with Tyr13 (Figure 6D). The N^2^ base (U in pri-let-7f1) undergoes stacking interactions with Trp40 (from β2), and N^3^ (G in pri-let-7f1) flips outwards and establishes polar interactions with Asn44 via its sugar 2’-hydroxyl group. Additionally, the flipped nucleotide base points into a negatively charged shallow groove on the Drosha belt-helix (formed by Glu822, Glu823, and Gln826). Interestingly, the RNA backbone at C^4^ kinks, which flips its base underneath the N^2^-N^3^ backbone and docks it into a positively charged shallow groove in the SRSF3 RRM domain formed by Trp40, Asn44, and Phe48, and forms a hydrogen bond between O2(C^4^) and the main chain amine of Arg43. Moreover, its sugar O1’ oxygen forms a H-bond with Asn44 (Figure 6D and S7C), stabilizing the kinked geometry. Beyond the CNNC motif, the RNA backbone further bends and stacks the −21 A base onto the N^2^ (U) base to further stabilize the CNNC fold (Figure S7A-B). The −22 U base further stacks onto the −21 A base without any direct contact with SRSF3 or Drosha.

Comparing the NMR structure of the SRSF3^RRM^-rCAUC tetranucleotide complex (Hargous et al., 2006) (PDBid 2I2Y) (Figure S7D) with the SRSF3-CNNC component in the MP^he^ bound structure reported here, revealed distinctive features in pri-miRNA recognition by SRSF3. In both cases, the third nucleotide of the motif is flipped out compared to the others. The C^1^ base interactions in both structures are nicely conserved. However, an interaction between Tyr13 and the C^1^ phosphate is observed in the pri-miRNA interaction, while the corresponding phosphate is absent altogether in the NMR structure. The N^2^ base recognition in pri-miRNAs is different as well. In the SRSF3 RRM-tetranucleotide structure Phe50 from β3 stacks onto N^2^ base, as is the case in almost all RRM-RNA complexes (Maris et al., 2005). However, this interaction is mediated by Trp40 from β2 when bound to the pri-miRNA (Figure S7C). In the SRSF3^RRM^-rCAUC structure, N^3^ (U) flips out and tucks into a shallow positive groove on the SRSF3 surface, H-bonds with Asn44, and no interactions with C^4^ were observed (Figure S7E-F). However, in the CNNC motif of pri-let-7f1, the flipped N^3^(G) is sensed by the Drosha belt. Notably, C^4^ docks into a positive pocket on the SRSF3 surface, with non-standard geometry. In contrast to the SRSF3 thumb peptide in the NMR structure, which does not interact with RNA, the thumb peptide in the pri-miRNA bound structure significantly swings onto the 3’ RNA strand enclosing the CNNC motif on top of the RRM domain (Figure S7A-B & S7D-E). Thus, the interactions between SRSF3 and its RNA binding site are influenced by its context.

To validate the importance of the observed interactions, we purified SRSF3 mutants that were designed to abolish interactions with either C^1^ and C^4^ or both (SRSF3-E79A/S81A or SRSF3-W40A/N44A, respectively) (Figure S5A). Figure 6E shows that in the *in-vitro* cleavage assay for pri-let-7c, much less of the productive cleavage product, pre-let-7c, is produced for the mutants compared with the reaction with WT-SRSF3 (Figure 6E **lane 3-6)**. This suggests that both C^1^ and C^4^ nucleotide recognition by SRSF3 are crucial for an effective CNNC motif-mediated response. We then tested the role of the thumb peptide in M^2^P^2^ of CNNC-containing pri-miRNAs using this assay. In contrast to the SRSF3 RRM (amino acids 1-84) domain alone, which produces a much-reduced level of the pre-let-7c product compared to the wild-type SRSF3, including the thumb peptide (amino acids 1-90) brought pre-let-7c product up to full-length levels (Figure 6E **lane 3, 8-9)**. This suggests that the thumb is crucial for productive processing of CNNC-containing pri-miRNA. The thumb peptide region is known to interact with NXF1/TAP (Hargous et al., 2006) during mRNA export, and our work reveals an additional role for this region in productive processing by the MP.

### The SRSF3 – Drosha interface

**Figure-7-:**
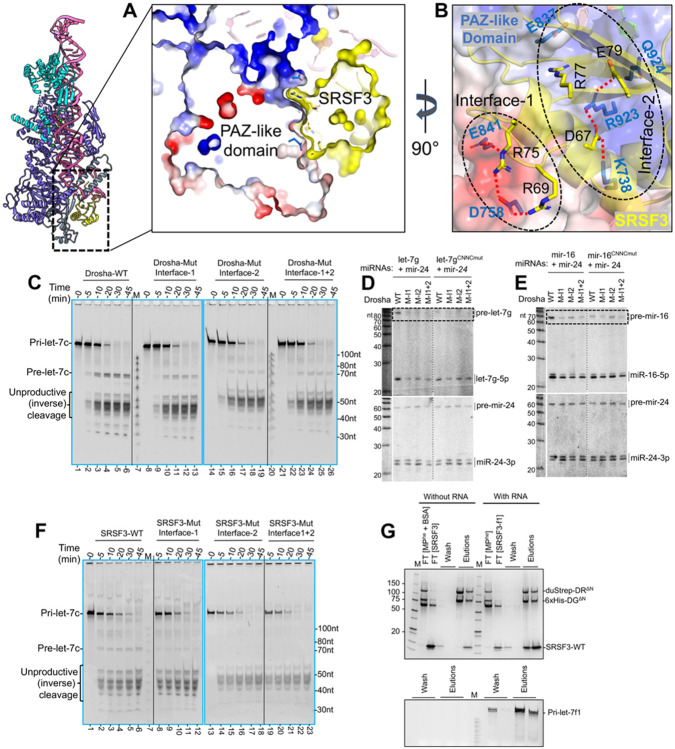
The SRSF3-Drosha PAZ-like domain interface is crucial for M^2^P^2^ of CNNC-containing pri-miRNAs. (A) A cutaway view of the Drosha-SRSF3 interface showing SRSF3 (yellow) perfectly nestled into the PAZ-like domain scaffold (shown as an electrostatic surface). Surface protrusions from SRSF3 are visible which fit into different cavities of the PAZ-like domain. (B) The SRSF3-PAZ-like domain interface is stabilized by several charged residues from SRSF3 (yellow sticks) and the PAZ-like domain (sky blue sticks), and clusters into two regions, interface-1 and interface-2 (black dotted ovals). (C) *In-vitro* M^2^P^2^ of pri-let-7c using Drosha-mutations in the Drosha-SRSF3 interface. Mutations in Interface-1, 2 or 1+2 show less or no pre-let-7c product during the time course. (D-E) Northern analysis of miRNA processing in HEK293T *Drosha-KO* cells rescued by wt or mutant Drosha constructs. Cells were co-transfected with expression constructs for CNNC-bearing pri-let-7g (D) or pri-miR-16 (E), or counterparts with mutations in CNNC motifs; non-CNNC pri-miR-24 serves as a negative control. Mutations in the SRSF3-interacting interface of Drosha reduce pri-miRNA processing for CNNC-bearing miRNAs, as clearly indicated by the reduction of pre-miRNA hairpin species (dotted boxes). These Drosha mutants also impaired accumulation of mature let-7g and miR-16 (asterisks), with a stronger effect on let-7g. Biogenesis of non-CNNC pri-miRNA constructs was not affected by Drosha mutations. (F) The Urea-PAGE showing *in-vitro* M^2^P^2^ of pri-let-7c with SRSF3-mutants at the Drosha-SRSF3 interface. (G) SRSF3 pull-down assay with MP^he^ and pri-let-7f1. SRSF3-WT co-elutes with the MP^he^-pri-let-7f1 as analyzed on SDS-PAGE (upper panel) and urea-PAGE (lower panel) gels.

The MP-SRSF3-pri-let-7f1 structure revealed a new protein-protein interface between SRSF3 and Drosha’s PAZ-like domain. The PAZ-like domain is essentially unchanged upon SRSF3 binding, implying that its surface is pre-formed for SRSF3 interaction. We should note, however, that this surface changed upon pri-miRNA binding. The PAZ-like domain exhibits a negative electrostatic potential on one side of its surface and a deep positively charged groove on the other, at the 3p RNA exit site (Figure S7G). Notably, the SRSF3 RRM exhibits the oppositely charged surfaces at its interface with the Drosha PAZ-like domain (Figure S7H) and docks perfectly onto that surface (Figure 7A). Therefore, there is both charge and surface complementarity between the two proteins.

From the positive protrusion on SRSF3, Arg69 and Arg75 establish a series of salt bridges with Asp758 and Glu841 from the PAZ-like domain, which we call “Interface-1”. For “Interface-2”, SRSF3 Asp67 and Glu79 form another set of salt bridges with Lys738 and Arg923 from the PAZ-like domain and wedge-loop, respectively. Additionally, SRSF3 Glu79 is stacked against Gln924 from the Drosha wedge further stabilizing the complex. SRSF3 Arg77 is stacked under Glu837 from the PAZ-like domain and points into a charged pocket near the 3’ RNA exit site, to establish polar interactions with Gly776 and Val838 main chain carboxyl group (Figure 7B).

To validate these interactions, we mutated interface-1 and interface-2 residues in SRSF3 and Drosha independently, and tested them in an *in-vitro* pri-let-7c cleavage assay with MP^he^. We designed mutations that reverse the charges to disrupt the specific interactions with the partner protein. We expressed and purified SRSF3 R69E/R75E and D67H/E79R/R77A as interface-1 and interface-2 mutants (Figure S5A), respectively, and Drosha interface-1 and interface-2 mutants D758H/E841R and K738E/R923E/Q924A, respectively. All purified mutant proteins eluted at the same retention volume as the respective WT proteins, indicating no problems with protein folding. Compared to WT MP^he^, the interface1 or interface-2 Drosha MP^he^ mutants result in significantly less pre-let-7c product formation in an *in-vitro* processing reaction (Figure 7C).

We further analyzed mutations in the SRSF3-interacting interface of Drosha and the requirement of pri-miRNA CNNC in cells. We rescued MP activity in *Drosha-KO* HEK293T cells by introducing wt or mutant Drosha constructs. At the same time, we co-transfected wt or CNNC-mutated miRNA constructs (let-7g or mir-16), along with the non-CNNC mir-24 as a control. Northern blotting showed that all three Drosha mutations compromised pri-miRNA processing, specifically for CNNC-bearing miRNAs. This was most clearly seen by the reduced levels of pre-let-7g and pre-miR-16 hairpins; mature let-7g was also strongly reduced while accumulation of mature miR-16 was impaired (Figure 7D-E). It is possible that perdurance of mature miRNAs obscures the effect of reduced nuclear cleavage of pri-miR-16. By contrast, CNNC-mutant pri-let-7g and pri-miR-16, along with non-CNNC pri-mir-24, were all equivalently processed by wt and Drosha variants bearing mutations at the SRSF3 interface (Figure 7D-E).

Pri-miRNA binding *in-vitro* for these mutated MP^he^ is largely unaffected compared to WT-MP^he^, (Figure S7I-J) suggesting that the observed effect is not due to impaired RNA binding. Similarly, compared to the WT-SRSF3, the SRSF3 mutations in interface-1 or interface-2 produced almost no pre-let-7c product in M^2^P^2^ (Figure 7F). To discern whether the reduced M^2^P^2^ of pri-let-7c in SRSF3 mutants is a consequence of their impaired Drosha PAZ-domain docking or an impaired RNA interaction, we performed *in-vitro* pull-down assays using the MP^he^-RNA as bait and gel-shift assay using pri-let-7 miRNAs. We found that the WT-SRSF3 co-elutes with the MP^he^-RNA complex (Figure 7G) and binds with the pri-miRNAs (Figure-S7K). Notably, both SRSF3 interface-1 and interface-2 mutants fail to co-elute with the MP^he^-RNA complex (Figure-S7L-N), while only the interface-2 mutant still binds the pri-miRNAs (Figure-S7K). These results suggest that the reduced M^2^P^2^ of pri-let-7c in interface-2 mutation is a direct consequence of impaired PAZ-domain docking. Overall, the mutation analysis supports the structural observation, that the interface between PAZ and SRSF3 is crucial for productive processing of pri-miRNAs.

## Discussion

Given the breadth of miRNA-mediated silencing in regulating gene expression across eukaryotes, accurate and efficient miRNA biogenesis is of premier interest. We have long been intrigued by the structural diversity of pri-miRNA substrates that the MP can tackle, even within the same miRNA family. Using the most abundant miRNA family in the human genome, the let-7 family, we investigated how MP recognizes and processes these diverse pri-miRNAs into pre-miRNAs.

In preparing the MP complex, we found that proper incorporation of heme affects processing whether or not a UGUG motif, previously implicated in heme recognition, is present in the pri-miRNA. Our structures showed that the heme-binding region, the WW motif of DGCR8, was, in fact, quite distant from the RNA and the UGUG motif, but might contribute to MP activity by promoting dimerization of DGCR8. We cannot rule out that dimerization might affect the interface between DGCR8 and the pri-miRNA apical loop, including the UGUG motif. However, this motif does not appear to interact with the HBD. In addition, Heme didn’t appear to have an effect on proper loading of the MP on the substrate, in contrast to previous suggestions (Partin et al., 2017).

We were fortunate that in this study we were able to observe the almost complete pri-miRNA, revealing the relative position of the apical-loop (preE) on top of the dsRNA stem (Figure 2B-D), in addition to the UGUG-motif, the GGAG-motif and the well-studied CNNC-motif (Figure 6). The pri-let-7’s apical loop in the different structures is exposed at least on one surface and could serve as a binding site for other RNA binding proteins (RBPs) to influence the M^2^P^2^ of specific pri-miRNAs, including Syncrip (Chen et al., 2020), Musashi-1 (Kawahara et al., 2011) and Lin28B (Viswanathan et al., 2008). Superimposing the crystal structure of Lin28a-let-7f1 preE (PDBid 5UDZ) (Wang et al., 2017) onto the MP^he^-pri-let-7f1 cryo-EM structure reported here revealed that the zinc-knuckle of Lin28 would clash with DGCR8 dsRBD’s binding to pri-let-7 (Figure-S3I) and might negatively impact M^2^P^2^ of pri-let-7s. While, the Lin28 cold-shock domain (CSD) would be positioned on the ss-loop away from DGCR8.

Let-7 pri-miRNAs segregate into two distinct classes based on whether a canonical 2-nt overhang is produced at the 3’-end for class-I pre-let-7s, or a single nt is left as the 3’ overhang in the class-II variety. It has been long speculated that a bulge at the 5p cleavage site is responsible for this difference. Here we show that the bulge alters the overall trajectory of the upper stem, essentially ‘straightening out’ the pri-miRNA (Figure 3C, S4D). In addition, the bulge perturbs the local RNA strand geometry (Figure 3K, S4E-G), allowing the following nucleotide to pair with the +3 nt on the 3p strand (Figure 3H-J). This alters the pairing register in the upper stem, resulting in a 1nt 3’ overhang following the cleavage reaction (Figure 3L-M). Quite often times, protein induces structural changes on the nucleic acids upon binding (Feng et al., 2021; Nikolov et al., 1995), but in M^2^P^2^ the RNA drives the repositioning of several domains of the enzyme as observed in different structures with class-I and class-II pri-let-7. (Figure 3D-F).

As mentioned, pri-miRNAs house a number of characteristic sequence motifs. We addressed two in our study. The first is the UG sequence motif at position −14 in the 5p strand, which we now suggest renaming the fUN (flipped U with paired N) motif. From our structural analysis of MP complexes with pri-miRNAs in the pre-catalytic state, we find that the U is unpaired in all cases we examined and is usually flipped (Figure 4, S4H-I). The role of the following nucleotide is to establish the first base pair in the lower stem. Though a G is more prevalent at this position in metazoan pri-miRNAs, and was shown to independently contribute to RNA catalysis (Auyeung et al., 2013), it appears to vary. The positioning of this motif is also slightly flexible and can be similarly accommodated at position −15.

The second motif is the CNNC motif at 3p −17 position that has a role in productive M^2^P^2^, mediated by a small splicing factor called SRSF3. SRSF3 is a major auxiliary factor in M^2^P^2^, modulating at least 84% of Drosha-dependent miRNAs (Kim et al., 2021). Our study shows how SRSF3 grabs onto the 3p strand of the RNA like a hand grabbing a rope at the CNNC motif (Figure 6A-B), between the thumb peptide and the RRM domain (Figure 6C). It is also tucked onto Drosha with complementing surfaces and electrostatics (Figure 7A-B). Thus, the MP-pri-miRNA complex is in a conformation that can bind SRSF3 without changes. SRSF3 situates Drosha firmly on the basal junction. It also prevents the ss region of the 3p strand from pairing with the 5p strand, which might extend the ds lower stem, as seen in pri-miR-16-1 (Jin et al., 2020), and could cause aberrant loading of the MP on the pri-miRNA.

SRSF proteins usually bind their target RNA through their RRM domain, while the RS-domain participates in protein-protein interaction (Manley and Krainer, 2010; Zhou et al., 2020). Though some RRM domains are known to directly interact with proteins via their α1 helix and β1-3 sheet (Kielkopf et al., 2004; Kielkopf et al., 2001; Selenko et al., 2003), SRSF3 RRM uses its α2-β4 side for Drosha interaction. The SRSF3 thumb-peptide region is known to interact with NXF1/TAP during mRNA export (Hargous et al., 2006), and the same region also interacts with the CNNC in M^2^P^2^, exhibiting its multifunctional nature.

Another splicing factor, the SR protein SRSF7 is also known to be involved in Drosha recruitment to the basal junction (Auyeung et al., 2013; Le et al., 2023). Its RRM shares ~73% identity with the SRSF3 RRM, with all residues involved in CNNC binding and Drosha PAZ-like domain interactions conserved in both proteins, suggesting a similar molecular mechanism for SRSF7.

Interestingly, MP^he^ pulls down SRSF3 in the absence of RNA (Figure 7G) and has a different sensitivity *in vitro* to the mutants we examined in the presence of RNA (Figure S7L-N). Since the surface of Drosha that interacts with SRSF3 changes upon binding the pri-miRNA (Partin et al., 2020), this implies that SRSF3 binds to a different surface of Drosha in the absence of RNA.

The MP-pri-mRNA structures reported here and elsewhere reveal MP’s invariable interactions with the lower stem of the RNA, measuring the distance from the basal junction to the cleavage sites. The presence of conserved sequence motifs in the lower stem does not appear to affect Drosha-lower stem geometry. This rigidity is complemented by its plasticity to reorient its dsRBDs with the upper stems of pri-miRNAs to accommodate a wide landscape of pri-miRNA structures. The combination of a rigid lower-stem length and interface, with the ability to reorient modules that could bind various characteristic motifs helps the MP distinguish these substrates from other structured RNAs.

## METHODS

### Experimental Models and Subject Details

The MuiltiBac baculovirus expression system was used for MP proteins expression in insect cells (Sf9 or HighFive). Baculovirus generated in Sf9 cells was maintained in HyClone CCM3 Cell Culture Media (Cytiva), while HighFive cells were cultured in ESF921 media (Expression systems).

### Expression and purification of human MP-heme variant proteins

Human Drosha isoform 4 and human DGCR8 clones were purchased from Addgene. cDNA encoding different length variants of Drosha were cloned into pFL plasmid and expressed as a N-terminal Dual-strep tag fusion, while DGCR8 cDNA length variants were cloned into pSPL and expressed as N-terminal 6xHis tag fusions. Different combinations of Drosha and DGCR8 clones were Cre-fused and expressed in either Sf9 or HighFive cells using the MultiBac baculovirus expression system. We used Drosha^317-1337^ and DGCR8^175-751^ for cryo-EM and biochemical studies. The insect cells were infected with baculovirus at 27°C for 60 hrs, and supplemented with 0.75 mM 5-aminoleuvelinic acid (5ALA) to enrich the MP protein with heme (MP^he^) during expression. Insect cells were harvested in resuspension buffer (50 mM Tris pH 8.0, 100 mM NaCl and 5 mM DTT) supplemented with protease inhibitor (PI) mix (Pepstatin, Leupeptin, PMSF, Benzamidine and Aprotinin) before flash freezing in liquid N2. The cells were thawed, 650 mM NaCl and 10% glycerol were added before sonication. After ultracentrifugation at 40,000 rpm for 1 hr, cleared lysate was loaded on 4 ml Strep-Tactin Superflow beads (IBA lifesciences) and washed extensively before co-eluting the Drosha and DGCR8 (MP) in 25 mM HEPES pH 7.5, 200 mM NaCl, 5 mM DTT, 10% glycerol supplemented with 7 mM desthiobiotin. The eluted MP protein was diluted with an equal volume of dilution buffer (50 mM Bis-Tris pH 6.8, 5 mM DTT and 10% glycerol) and loaded onto the HiTrap SP HP cation exchange column (Cytiva) pre-equilibrated in Buffer-A (25 mM Tris pH 6.8, 75 mM NaCl, 5 mM DTT and 10% glycerol). A linear NaCl gradient (from 75 mM to 1 M) was used to elute the MP.

Heme-free MP (MP^apo^) and heme-bound MP (MP^hb^) had different ionic strengths and were separated during HiTrap SP-HP column chromatography, as observed from Abs450 peak and A450/280 ratios. The heme-enriched MP (MP^he^) protein eluted as a single peak with a higher Abs450 and A450/280 ratio. Eluted heme-variant MP proteins were separated, analyzed on SDS-PAGE, pooled and loaded onto Superose 6 increase 10/300 gel filtration column (Cytiva) pre-equilibrated with SEC buffer (25 mM HEPES pH 7.5, 400 mM NaCl, 5 mM DTT and 10% glycerol). Peak fractions for eluted MP proteins were concentrated and flash frozen in liquid N_2_ and stored at −80°C

### Expression and purification of SRSF3 proteins

The human SRSF3-pET28a clone was purchased from Genscript (Piscataway, NJ). Different SRSF3 variants were subcloned in pET28a and expressed as TEV protease-cleavable N-terminal 6xHis-tag fusions in *E.coli* Rosetta2(DE3) cells (Novagen). *E.coli* cells were grown in TB media up to an OD_600_ of ~ 1.2 at 37°C, and protein expression was induced for 16 hr at 18°C by adding 0.5 mM IPTG. Cells were harvested in *E.coli* resuspension buffer (25 mM Tris pH 8.0, 500 mM NaCl, 5 mM ß-Me and 10 mM Imidazole), supplemented with PI cocktail (pepstatin, leupeptin, PMSF, benzamidine and aprotinin) before flash freezing in liquid N_2_. The cells were thawed and lysed by sonication and bulk nucleic acids were precipitated by mixing 0.2% PEI (Poly-ethylenimine) into the lysate before ultracentrifugation at 40,000 rpm for 1 hr. Using the cleared lysate, Ni-NTA affinity chromatography was performed to elute 6xHis-SRSF3 protein in Ni-NTA elution buffer (25 mM HEPES pH 7.5, 200 mM NaCl, 5 mM ß-Me, 250 mM imidazole and 5% glycerol). The eluted protein was diluted to reduce the NaCl concentration to 50 mM using dilution buffer (25 mM HEPES pH 7.4, 5 mM ß-Me and 5% glycerol) and loaded onto HiTrap SP-HP column (Cytiva) pre-equilibrated with cation exchange buffer-A (25 mM HEPES pH 7.4, 50 mM NaCl, 5 mM ß-Me and 5% glycerol). NaCl linear gradient from 50 mM – 1000 mM was used to elute the 6xHis-SRSF3 protein and analyzed on SDS-PAGE. Fractions containing pure protein were pooled, concentrated and injected into the Superdex 75 increase 10/300 column (Cytiva) pre-equilibrated with SEC buffer (25 mM HEPES pH 7.5, 150 mM NaCl and 1 mM DTT). Purified SRSF3 protein was concentrated before flash freezing in liquid N_2_.

### MicroKit SEC for MP heme-variants

Different MP heme-variants were analyzed by injecting 2.4 μM (12 μg total protein) of purified protein into Superose 6 increase 3.2/300 column (Cytiva) pre-equilibrated in Microkit SEC buffer (HEPES 25 mM pH 7.5, NaCl 400 mM, DTT 5 mM, 10% glycerol and 10 μM ZnCl_2_). Absorption at UV280, UV260 and UV450 were recorded, while UV280 and UV450 traces were used to plot the final chromatograms. The A450/280 ratio was calculated from the values at the tip of each chromatogram peak. N=3.

### In-vitro transcription (IVT) of pri-miRNAs

The cDNAs encoding different pri-let-7 miRNAs were subcloned in pRSF plasmid, with a flanking 5’ hammerhead (HH) ribozyme and 3’ hepatitis delta virus (HDV) ribozyme sequences. Large scale *in-vitro* transcription reactions were run using in-house purified T7 RNA polymerase. After transcription, DNA templates were digested using RNase-free DNase (NEB), and RNA was phenol-chloroform extracted followed by isopropanol precipitation (overnight at −80°C). The RNA pellet was dissolved in TE buffer, and PAGE purified from a 6% denaturing polyacrylamide gel in RNase-free water. Purified pri-miRNA was concentrated using Amicon Ultra-14 (10 kDa MWCO) and stored at −80°C.

### Pri-miRNA cleavage assays

*In-vitro* pri-miRNA time course cleavage assays were performed by incubating ~150 nM pri-miRNA with ~300 nM purified MP protein in processing assay buffer (20 mM HEPES pH 7.5, 100 mM NaCl, 2.5 mM DTT, 5 mM MgCl_2_, 2 U/ml RNasin and 10% glycerol) at a final volume of 100 ul. The reaction mix was incubated at 37°C and 15 ul aliquots were removed at indicated time points, quenched into stop buffer (1.8% SDS, 10 mM EDTA) followed by Proteinase-K (2 units total) (NEB) treatment for 45 min at 50°C. The samples were diluted with the 2X denaturing buffer (80 % formamide, 1.5 M urea, 2 mM EDTA, 0.05% bromophenol-blue and 0.05% xylene-cyanole) and analyzed on a 12% urea-PAGE (20 cm X 20 cm) (National diagnostics). Gels were stained with SybrGold stain and visualized on BioRad ChemiDoc imaging system.

For RNA processing assays with SRSF3, ~150 nM pri-miRNA was first incubated with 350 nM SRSF3 protein for 15 min at 30°C in processing assay buffer. Catalysis was initiated by adding ~250 nM MP protein, and 15 ul aliquots were removed at indicated time points for analysis.

### Quantitative biochemical analysis for pri-let-7 processing

*In-vitro* synthesized pri-miRNAs (100 pmol) were 5’-end labelled by T4-PNK (NEB) using [γ-^32^P]-ATP (Perkin Elmer) and purified using a MicroSpin G-25 column (Cytiva). The labelled RNAs were further extracted by the phenol:chloroform method and precipitated using isopropanol for 15 mins at −80°C. The RNA pellet was washed with 80% ethanol and dissolved in 20 ul RNA labelling buffer (50 mM MOPS, pH 6.0, 50 mM NaCl, 0.1 mM EDTA). The absolute RNA concentration was measured by scintillation.

For biochemical assays, 100 ul RNA catalysis reactions were run at 30°C with MP variants pre-incubated in processing assay buffer (20 mM HEPES pH 7.5, 100 mM NaCl, 2.5 mM DTT, 5 mM MgCl_2_, 1 U/ml RNasin and 10% glycerol) for 5 mins. Catalysis was initiated by adding 1 nM of 5’ ^32^P-labelled pri-miRNA. 10 ul aliquots were removed at 0.16’, 0.5’, 1’, 2’, 5’, 10’, 15’, 30’, 60’ and 90’ and directly quenched into 2 ul of 5X stop buffer (9% SDS and 50 mM EDTA). The samples were further treated with 2U Proteinase-K (NEB) for 45 min at 50°C before diluting the samples with 2X denaturing buffer (80 % formamide, 1.5 M urea, 2 mM EDTA, 0.05% bromophenol-blue and 0.05% xylene-cyanole). Samples were heated at 95°C for 5 mins before loading 12 ul onto the 12% denaturing urea-PAGE (National diagnostics).

For the biochemical assays involving SRSF3, 1 nM of 5’ ^32^P-labelled pri-miRNA was pre-incubated with 2.5 nM SRSF3 for 15 mins at 30°C in processing assay buffer. The catalysis was initiated by adding MP^he^ protein, and 10 ul aliquots were removed at 0.16’, 0.5’, 1’, 2’, 5’, 10’, 15’, 30’, 60’ and 100’ and processed as described above. MP^he^ concentrations of 0.3 nM and 0.6 nM were used for pri-let-7c and pri-let-7a1, respectively, which were optimized to observe a complete RNA cleavage and clear difference in RNA cleavage in different conditions.

Gels were exposed to phosphor screens overnight and imaged using Typhoon FLA 7000 (GE healthcare Life Sciences). Bands corresponding to leftover pri-miRNA substrate, generated pre-miRNA (productive cleavage) and the unproductive cleavage product were quantified using ImageQuant7 (GE healthcare) and values normalized for each lane.

GraphPad Prism 9 was used for data analysis, and statistical analysis. Quantified data could be fitted to a bi-phasic decay equation, to calculate the observed rate constants (K_obs_) for two phases of decay at different MP concentrations (K_obs_^fast^ and K_obs_^slow^). The margin of error (MOE) = [upper limit – lower limit] / 2.

### Electrophoretic Mobility Shift assays (EMSA)

For EMSA, ~150 nM pri-let-7 RNA was mixed with different MP^he^ proteins in a 1:1, 1:2 and 1:4 molar ratios in EMSA buffer-1 (25 mM HEPES pH 7.5, 100 mM NaCl, 2.5 mM DTT, 5 mM CaCl_2_, 10 % glycerol, 0.01 % Triton X-100 and 1 U/ml RNasin). The reaction was incubated on ice for 1 hr, 20 % glycerol was added and analyzed on a 5 % TBE gel (Bio-Rad) run in ice-cold 0.5 X TBE buffer. Gels were stained with SybrGold and visualized on BioRad ChemiDoc imaging system.

For EMSA with SRSF3, 1 nM of the 5’ ^32^P-labelled pri-miRNA was incubated with SRSF3 proteins at the indicated concentrations in EMSA buffer (25 mM HEPES pH 7.5, 75 mM NaCl, 2 mM DTT, 8% glycerol, 0.01 % Triton X-100, 1 mM EDTA, 0.1 mg/ml BSA) for 1 hr at 4°C. EMSA loading dye (50 % glycerol, 0.05 % xylene cyanol and 0.05 % bromophenol blue) was added and samples analyzed on a 20x20 cm 4 % TBE gel run in ice-cold 0.5 X TBE buffer. Gels were exposed to phosphor screens overnight and imaged using Typhoon FLA 7000 (GE healthcare Life Sciences).

### In-vitro pull-down assays

Pull-down assays were performed with MP^he^, pri-let-7f1 against different SRSF3 proteins (WT or mutants as marked in the figures). 25 μl Strep-Tactin beads were coated with BSA (0.2 mg/ml) for 20 mins, followed by incubation with 4 μM MP^he^ for 15 mins on ice. The mixture was centrifuged at 200 xg for 4 min, and excess solution (FT-MP^he^+BSA) was removed. Beads were then incubated with 20 μM of different SRSF3 protein with or without 6 μM of pri-let-7f1 for 20 mins on ice. The flow-through (FT- SRSF3-f1) was collected before sequentially washing the beads with 200 μl, 400 μl & 500 μl wash buffer (25 mM HEPES pH 7.5, 200 mM NaCl, 2 mM DTT, 5 mM CaCl_2_, 10% glycerol, 0.01% Triton X-100). Bound complexes were eluted twice in 30 μl elution buffer (25 mM HEPES pH 7.5, 100 mM NaCl, 2 mM DTT, 5 mM CaCl_2_, 10% glycerol, 0.01% Triton X-100 and 5 mM desthio-biotin) and analyzed on SDS-PAGE and urea-PAGE.

### Northern blots

Co-transfection of the Drosha-KO HEK293T cells (Shang and Lai, PNAS, 2023) with miRNA plasmids (for each well: 300 ng of WT or CNNC mutant miRNAs with 50 ng of non-CNNC mir-24 plasmids) and Drosha plasmids (800 ng per well) was performed in 12-well cell culture plates using Lipofectamine2000 (Thermo Fisher) according to the manufacturer’s protocol. Cells were harvested 48 hours post-transfection using Trizol reagent for total RNA extraction. Equal amounts of total RNAs (10 μg) were denatured at 95°C and fractionated by electrophoresis on a 20% urea polyacrylamide gel in 0.5x TBE buffer. The gel was transferred to GeneScreen Plus membrane (Perkin Elmer) at 300 mA for 1.5 hr, UV-crosslinked and baked at 80°C for 30 min and hybridized with γ-^32^P-labeled miRNA probes at 42°C overnight (hybridization buffer: 5x SSC, 7% SDS, 2x Denhardt’s solution). The membrane was washed with Non-Stringent Wash Solution (3x SSC, 5% SDS, 10x Denhardt’s solution) followed by two rounds of wash with Stringent Wash Solution (1x SSC, 1% SDS). Each wash step was conducted at 42°C for 30 min. The membrane was sealed in plastic wrap, inserted into a film cassette and exposed for 1~3 days. For re-probing of the same blot with other miRNA probes, the blot was washed with 1% SDS at 80°C for 30 min and then hybridized with the following miRNA probes: anti-mir-16-5p CGCCAATATTTACGTGCTGCTA; anti-let-7g-5p AACTGTACAAACTACTACCTCA; anti-mir-24-3p CTGTTCCTGCTGAACTGAGCCA.

### Cryo-EM sample preparation

Pri-let-7s were annealed by heating at 85°C for 5 min and snap cooling on ice for 10 mins. MP^he^ and different pri-let-7s were mixed in 1:1.25 molar ratios in cryo-EM reaction buffer (25 mM HEPES pH 7.5, 100 mM NaCl, 5 mM DTT, 5 mM CaCl_2_) and incubated for 1:30 hr on ice. The complex was crosslinked using 2 mM DSG (ThermoFisher Scientific) for 25 mins at 20°C. The crosslinked complex was separated from excess RNA via SEC over Superose 6 increase 10/300 column (Cytiva) pre-equilibrated in cryo-EM sample buffer (20 mM HEPES pH 7.5, 120 mM NaCl, 5 mM DTT, 5 mM CaCl_2_) and concentrated to ~1.0 – 1.5 mg/ml (Bradford method) before grid preparation. For MP^he^-pri-let-7f1-SRSF3 sample, 1.4 fold molar excess SRSF3-FL to RNA was added while assembling the complex before crosslinking. For MP^he^-pri-let-7a2 sample, SRSF3-RRM was added in the sample, but no density was observed for the SRSF3-RRM domain.

For cryo-EM grid preparation, 0.05% w/v β-OG (Octyl β-D-glucopyranoside) (ThermoFisher Scientific) detergent were added prior to application of 3 μl sample onto a glow-discharged Quantifoil R 0.6/1 300 mesh grid (for MP^he^-pri-let-7a1, MP^he^-pri-let-7f1, MP^he^-pri-let-7f1-SRSF3) or glow-discharged UltrAuFoil R 0.6/1 300 mesh grid (for MP^he^-pri-miR-98, MP^he^-pri-let-7a2, MP^he^-pri-let-7f1-SRSF3), incubated for 10 sec at 25°C and 95% humidity, blotted for 3.1 s, and plunged into liquid ethane using an Automatic Plunge Freezer EM GP2 (Leica).

### Cryo-EM data acquisition

Cryo-EM data were collected on a Titan Krios transmission electron microscope (ThermoFisher Scientific) operating at 300 keV. EPU data collection software (v 2.10.0.5) (ThermoFisher Scientific) was used, and dose-fractionated movies were collected using a K3 direct electron detector (Gatan) operating in electron counting mode.

For MP^he^-pri-let-7a1, 30-framed movies were collected with an exposure rate of 2.405 e^−^/Å^2^/frame resulting in a cumulative exposure of 72.16 e^−^/Å^2^. A total 11,160 micrographs were collected at 81,000x magnification (1.1 Å/pixel) and defocus range of 0.6 to 2.2 μm. For MP^he^-pri-miR-98, 30-framed movies were collected with an exposure rate of 1.42 e^−^/Å^2^/frame resulting in a cumulative exposure of 42.6 e^−^/Å^2^. A total 11,051 micrographs were collected at 81,000x magnification (1.1 Å/pixel) and defocus range of 0.7 to 2.2 μm. For MP^he^-pri-let-7a2, 30-framed movies were collected with an exposure rate of 2.6 e^−^/Å^2^/frame resulting in a cumulative exposure of 78 e^−^/Å^2^. A total 5,486 micrographs were collected at 105,000x magnification (0.856 Å/pixel) and defocus range of 0.6 to 2.0 μm.

For MP^he^-pri-let-7f1, 30-framed movies were collected with an exposure rate of 2.59 e^−^/Å^2^/frame resulting in a cumulative exposure of 77.6 e^−^/Å^2^. A total 8,217 micrographs were collected at 105,000x magnification (0.856 Å/pixel) and defocus range of 0.6 to 2.2 μm in CDS (correlative double sampling) mode.

For MP^he^-pri-let-7f1-SRSF3, 30-framed movies were collected with an exposure rate of 2.55 e^−^/Å^2^/frame resulting in a cumulative exposure of 76.6 e^−^/Å^2^. Total 7,995 and 6,429 micrographs were collected at 105,000x magnification (0.856 Å/pixel) and defocus range of 0.7 to 2.2 μm from a QuantiFoil and UltrAuFoil grid respectively.

### Cryo-EM Image processing

WARP (v 1.0.9) was used for real-time image pre-processing (motion correction, CTF estimation, and particle picking) (Tegunov and Cramer, 2019) for all the MP^he^-pri-let-7 miRNA structures. Particle picking was performed with the BoxNet pretrained neural network bundle included in WARP that is implemented in TensorFlow. A particle diameter of 180 Å and threshold score of 0.6 yielded 1,749,636 particle coordinates for MP^he^-pri-let-7a1, 967,368 particle coordinates for MP^he^-pri-miR-98, 1,742,639 particle coordinates for MP^he^-pri-let-7f1, 771,705 particle coordinates for MP^he^-pri-let-7a2. For MP^he^-pri-let-7f1-SRSF3, WARP processing yielded 846,678 and 871,735 particle coordinates for the QuantiFoil and UltrAufoil datasets respectively. All subsequent processing steps were carried out in cryoSPARC v3.2 (Punjani et al., 2017).

For all the structures, extracted particles were 2D classified, and a subset of those were used for ab-initio 3D reconstruction after manually inspecting each 2D class. Separating particles into total a 8-10 ab-initio classes was critical in improving the map quality for all the structures in this study. The resulting models were then used for 3D heterogeneous refinement against the whole particle set. Thus, we separated 397,113 particles for MP^he^-pri-let-7a1 (Figure S2D), 209,910 particles for MP^he^-pri-miR-98 (Figure S2A), 459,947 particles for MP^he^-pri-let-7f1 (Figure S2G) and 251,413 particles for MP^he^-pri-let-7a2 (Figure S4A) for further refinements. For MP^he^-pri-let-7f1-SRSF3 3D classification yielded 254,462 and 234,705 particles from the QuantiFoil and UltrAuFoil datasets respectively (Figure S6A).

For MP^he^-pri-let-7a1, 397,113 particles were refined with 4 random decoy classes, followed by a homogeneous and a non-uniform refinement yielding a cryo-EM map of 3.2 Å resolution according to gold standard FSC (GSFSC) (Figure S2E).

For MP^he^-pri-miR-98 and MP^he^-pri-let-7f1, 3D classified particle stacks (209,910 and 459,947 particles respectively) were iteratively processed by 2 cycles of heterogeneous and homogeneous refinement separating 173,800 and 378,162 particles for their respective structures. This particle stack was non-uniformly refined to generate 3.2 Å and 2.9 Å resolution cryo-EM maps (according to GSFSC) for MP^he^-pri-miR-98 (Figure S2B) and MP^he^-pri-let-7f1 (Figure S2H) respectively.

Similarly, for MP^he^-pri-let-7a2, one homogeneous and non-uniform refinement of 251,413 particles yielded the final 2.9 Å cryo-EM map according to GSFSC (Figure S4A-B). For MP^he^-pri-let-7f1-SRSF3, the two 3D refined particle stacks were merged (total 489,167 particles) and taken for 2 cycles of iterative heterogeneous and homogeneous refinements separating 325,534 good particles. Finally, a non-uniform refinement was performed to generated cryo-EM map of 3.1 Å resolution, according to GSFSC (Figure S6).

We further performed local sharpening and de-noising of cryo-EM maps using non-linear post-processing with Deep cryo-EM Map Enhancer (DeepEMhancer) (Sanchez-Garcia et al., 2021). These DeepEMhanc’ed maps had much improved cryo-EM density for Drosha, DGCR8, and the apical loop of the RNA (Figure S2C, S2F, S2I). Sharpened maps were used for model visualizations and building, while original maps were used for structure model refinements.

### Model-building, refinement and validation

The atomic model of MP-pri-miR-16-2 (PDBid 6V5B) (Partin et al., 2020) was used as a starting model for the MP^he^-pri-miR-98 structure, which was rigid body fitted in the map using ChimeraX (Pettersen et al., 2021). Although Drosha (from MP-pri-miR-16-2) aligned well in the cryo-EM map, several regions had to be rebuilt to get the final MP^he^-pri-miR-98 structure model (Figure 2C), which was then used as template for the other MP^he^-let-7 structures in this study. Though the nucleotide register was easily identifiable in the maps, we also utilized the M-fold server (Zuker, 2003) as a guide for building a few base-pairs in RNA upper stem only. The atomic model of the let-7 pre-miRNA apical loop from the Lin28-pre-let-7 crystal structure (PDBid 5UDZ) (Wang et al., 2017) was fitted into the MP^he^-pri-let-7a1 map using ChimeraX, and individual nucleotides were replaced to match the appropriate sequence in Coot (v 0.9.4) (Emsley and Cowtan, 2004). For the MP^he^-pri-let-7f1-SRSF3 structure the extended 3p RNA fragment was manually build using Coot.

The atomic model of the SRSF3 RRM domain (PDBid 2I2Y) (Hargous et al., 2006) was used to rigid body fit into the map density near the Drosha PAZ-like domain using ChimeraX and manually build further. All atomic model building was done in Coot, and refinements were performed in PHENIX (v1.20.1-4487-000) (Adams et al., 2010; Liebschner et al., 2019). Secondary structure restraints for protein and RNA were used throughout the refinement process. The DeepEMhance’d maps were also utilized for visualizing and building the structure models, while we used the unsharpened cryo-EM map for refinements. Structure validation was done using the MolProbity server (Chen et al., 2010). The structure figures were generated by ChimeraX and PyMOL molecular graphics system (Version 2.5.5, Schrödinger, LLC, Heidelberg, D).). The data collection and model statistics are summarized in Table S1.

### Lead contact and Material availability

Further information and requests for resources and reagents should be directed to and will be fulfilled by the Lead Contact, Leemor Joshua-Tor (leemor@cshl.edu). Reagents generated in this study are available from the Lead Contact with a completed Materials Transfer Agreement.

### Limitation of the study

Though we can resolve more of the HBD of Drosha that was reported previously, a significant portion of that domain is still untraceable. In addition, we could not identify the heme itself. Therefore, the interplay between heme and the UGUG motif, in particular, needs further characterization. Though a good representation of let-7 pri-miRNAs were used in this study, other pri-miRNAs may add to a more complete understanding of how the MP recognizes pri-miRNAs and how that may affect M^2^P^2^.

## Supplementary Material

Supplement 1
