# Supplementary material for: The structural landscape of Microprocessor Mediated pri-let-7 miRNAs processing
Source: bioRxiv. 2024 May 9:2024.05.09.593372. Preprint. [Version 1] doi: 10.1101/2024.05.09.593372 (PMC11100773; doi:10.1101/2024.05.09.593372)
Supplement: Supplementary file 1 [file media-1.pdf]

Supplemental figures and tables

The structural landscape of Microprocessor Mediated pri-*let-7* miRNAs processing

Ankur Garg[1, 2], Renfu Shang[3], Todor Cvetanovic[1], Eric C. Lai[3]
and Leemor Joshua-Tor[1, 2*]

[1] W. M. Keck Structural Biology Laboratory, Cold Spring Harbor Laboratory, One Bungtown Road, Cold Spring Harbor, New York, 11724 USA

[2] Howard Hughes Medical Institute, Cold Spring Harbor laboratory, One Bungtown Road, Cold Spring Harbor, New York, 11724 USA

[3] Developmental Biology Program, Sloan Kettering Institute, 430 East 67th St, ROC-10, New York, NY 10065, USA

* Correspondence to: leemor@cshl.edu

Supplementary Figure Legends

Figure-S1- MP heme-variants (MP^{apo}, MP^{hb} and MP^{he}) and *in-vitro* M^2P^2 of pri-let-7s.

(A) Cation exchange chromatogram for MP protein expressed without 5ALA showing separation of heme-bound MP (MP^{hb}) and heme-free MP (MP^{apo}) fractions as observed by UV450 absorbance. The separated MP^{hb} and MP^{apo} peaks show homogeneous proteins during size exclusion with average A450/280 ratios of ≤ 0.20 and < 0.10 respectively.

(B) Cation exchange chromatogram of MP expressed with 5ALA. Heme-enriched MP (MP^{he}) eluted as a single peak with an average A450/280 ratio of ≥ 0.25. SDS-PAGE gel showing the purified MP^{he} protein used in this study ($DR^{\Delta N}DG^{\Delta N}$).

In-vitro processing assay for different pri-let-7 miRNAs by (C) MP^{he} and (D) MP^{apo}. For most RNAs, MP^{he} generated a clear pre-miRNA species in a 30-min time course assay. Pri-let-7c is inversely processed by MP^{he} generating a minimal amount of pre-let-7c. MP^{apo} produces significantly less pre-let-7s products compared to MP^{he}. Different RNA species observed are marked. The 100 nt RNA ladder is denoted by “M”.

Figure-S2- Cryo-EM structures for class-II pri-let-7s (miR-98, a1 and f1) miRNA in complex with MP^{he}.

The cryo-EM data processing flow chart for (A) MP^{he}-pri-miR-98 (D) MP^{he}-pri-let-7a1 and (G) MP^{he}-pri-let-7f1 structures. Extracted particles were sorted during ab-initio 3D reconstruction and map quality was further improved by iterative rounds of heterogeneous and homogeneous refinement, followed by non-uniform refinement (Punjani et al., 2020). The local resolution map and GSFSC resolution estimate for (B) MP^{he}-pri-miR-98 (E) MP^{he}-pri-let-7a1 and (H) MP^{he}-pri-let-7f1 cryo-EM maps. The Drosha-RNA stem region is resolved to higher resolution than other parts of the complex. DGCR8 dsRBDs are at ~4-5 Å resolution, while HBD is resolved to ~6-7 Å.

Comparisons of the cryo-EM maps processed with and without DeepEMhancer are shown for (C) MP^{he}-pri-miR-98 (F) MP^{he}-pri-let-7a1 and (I) MP^{he}-pri-let-7f1. The overall map density is improved for DeepEMhance’d maps. Both the RNA register and the apical loop position are identifiable in the density modified maps.

Figure-S3- MP^{he}-pri-let-7 miRNA complex provides insights into complex assembly and RNA catalysis.

(A-B) The MP^{he}-pri-let-7a1 structure overlaid with the DeepEMhancer modified cryo-EM map, revealing density for the HBD. The WW motif crystal structure (PDBid 3LE4) (purple) is docked into the map. The structural features for the HBD are partially observed but are not modelled in the structure.

(C-D) The AlphaFold-multi (Jumper et al., 2021) predicted HBD model fit into the cryoEM map corresponding to the HBD dimer. Only the WW motif dimer fits reasonably well, but other structural features do not.

The cryo-EM density corresponding to the 5' catalytic site of (E) MP^{he}-pri-let-7a1, (F) MP^{he}-pri-let-7f1 (G) MP^{he}-pri-miR-98 and (H) MP^{he}-pri-let-7a2 maps showing the conserved water molecules (red) and Ca^{2+} ion (green). The water coordinating residue Q1144, and Ca^{2+} interacting residue E1222 are shown as sticks.

(I) Structural superposition of the MP^{he}-pri-let-7f1 apical loop region (pink) with the crystal structure of the Lin28a-let-7f1 apical loop (grey). The zinc-knuckle of Lin28 (shown as a yellow surface) would clash with the DGCR8 dsRBD's (cyan cartoon). The Lin28 CSD is shown as a green surface.

Figure-S4- Structural analysis of class-I and class-II pri-let-7.

(A) Cryo-EM data processing flow chart for MP^{he}-pri-let-7a2 (class-I) complex structure. Selected 2D classes are also shown. (B) Local resolution map and GSFSC resolution estimation plot for MP^{he}-pri-let-7a2. (C) Side-by-side comparisons of the cryo-EM map and DeepEMhancer modified map for MP^{he}-pri-let-7a2 structure.

(D) Superposition of the class-I and class-II pri-let-7s miRNA bound to MP^{he} in the pre-catalytic state. The positioning of class-I and class-II pri-let-7 upper stems are different, which is indicated by the black arrow. pri-let-7a2 belongs to class-I let7s.

(E) The difference in the backbone geometry in class-I and class-II pri-let-7s, imposed by the 5p +1 bulge nucleotide in 5' cleavage site. The 5p +2 nt (green) is pushed outward in class-II pri-let-7s (7a1, 7f1, miR98) changing the backbone direction. The cleavage sites are marked with red arrows.

The (F) shear and (G) step rise parameters for basepairs around the 5' cleavage site in different pri-let-7s. The basepair involving the 5p +2 nt shows significant changes compared to others (red).

A zoomed-in view of interactions observed for canonical 5' UG motif in pri-miR-16-1 (H) and non-UG motif in pri-miR-16-2 (I). Different residues interacting with RNA nucleotides are shown as sticks (green).

Figure-S5- SRSF3 assists in M^2P^2 of CNNC motif containing pri-miRNA.

(A) SDS-PAGE gel showing purified SRSF3 proteins, mutants/truncations used in this study. SRSF3-E79A/S81A and SRSF3-W40A/N44A correspond to SRSF3-FL with mutations in residues recognizing C^1 and C^4 nucleotides in the CNNC motif, respectively. SRSF3 RRM and RRM-thumb represents the $SRSF3^{1\text{-}84}$ and $SRSF3^{1\text{-}90}$ truncations. SRSF3-R69E/R75E and SRSF3-D67H/E79R/R77A are SRSF3-FL with mutations in interface-1 and interface-2 of the Drosha-PAZ like domain, respectively. Purified MP^{he} ($DR^{\Delta N}DG^{\Delta N}$) protein is shown in lane 1. The protein ladder is marked as "M".

(B) Processing assay for pri-let-7c in the presence of SRSF3 showing productive cleavage products (pre-let-7c) (also in C). Almost no pre-let-7c is produced in the absence of SRSF3 (lane 9), Addition of 5 mM Ca^{2+} inhibits the reaction. Unproductive cleavage products are marked. (C) The RRM domain fails to generate pre-let-7c. No effect is observed in the catalysis of CNNC-deficient pri-let-7a1 in the presence of SRSF3-FL. The 100nt RNA ladder is marked as "M".

(D) *In-vitro* processing assays of CNNC motif containing pri-let-7g. Urea-PAGE gel analysis shows less unproductive cleavage products upon SRSF3 addition (lane-8-12) (green arrows). Pri-let-$7g^{CNNC/UUUU}$ mutant shows a higher level of unproductive cleavage (lanes 15-19) (red arrows). An effect on productive cleavage (pre-let-7g) is not very apparent. The 100nt RNA ladder is marked as "M". Contaminant ribozyme in the pri-let-7g RNA prep is marked and is not catalyzed by MP^{he}.

Figure-S6- The cryoEM structure of MP^{he}-pri-let-7f1-SRSF3

(A) The cryo-EM data processing flow chart for MP^{he}-pri-let-7f1-SRSF3 quaternary structure. Two collected cryo-EM datasets were separately processed and 3D

heterogeneously filtered particles from both datasets were merged for further processing. The iterative heterogeneous refinement followed by non-uniform refinement resulted in the final map. Selected 2D classes from each dataset are shown.

(B) Cryo-EM map of MP^{he}-pri-let-7f1 and (C) MP^{he}-pri-let-7f1-SRSF3 show extra density near the Drosha-belt-helix (red dotted oval). The map quality significantly improved after (D) DeepEMhancer processing. (E) Local resolution map and (F) GSFSC resolution estimation plot for MP^{he}-pri-let-7f1-SRSF3 map.

Figure-S7- SRSF3 presents complimentary surfaces for CNNC binding and Drosha PAZ-like domain docking.

Comparison of the cryo-EM SRSF3-pri-let-7f1 structure with the SRSF3-rCNNC tetranucleotide NMR structure (PDBid 2I2Y). Shown are the cartoon (A & D), electrostatic surface (B & E) and stick (C & F) representations for the respective structures. For the quaternary structure, only 7 terminal nucleotides of the 3p strand are shown. The CNNC motif is colored green. The "thumb" (cyan) is in an open conformation (D-E) and closes on top of the CNNC in the MP-pri-miRNA complex (A-B). The C^1, N^2, N^3 and C^4 nucleotides have different interaction patterns in the two structures.

(G) Electrostatic surface of the Drosha PAZ-like domain with interacting SRSF3 residues shown as yellow sticks and interacting residues from Drosha as blue sticks. (H) The electrostatic surface of SRSF3 with interacting residues from the Drosha PAZ-like domain shown in blue sticks and SRSF3 residues in yellow. Both proteins show complementary positive and negative patches on their surface, which dock onto each other to create the Drosha-SRSF3 interface (black dotted ovals).

Gel-shift assay analyzing the (I) pri-let-7a1 and (J) pri-let-7c binding of MP^{he} with Drosha mutations in SRSF3 interacting interfaces. Interface1 or 2 mutations are marked as $DR^{\Delta N}$-Mut-I1 and $DR^{\Delta N}$-Mut-I2, respectively.

(K) EMSA comparing pri-let-7 binding of SRSF3 with mutations in its Drosha-binding interface. SRSF3 concentrations are marked atop each lane.

(L-N) Pull-down assays of SRSF3 mutants with the MP^{he} and pri-let-7f1. SRSF3-R69E/R75E and SRSF3-D67H/E79R/R77A correspond to residues involved in interactions with Drosha at interface1 and interface2, respectively. SDS-PAGE gel (upper

panel) shows that SRSF3 mutants do not co-elute with the MP^{he}-pri-let-7f1 complex. Urea-PAGE gel (lower panel) shows the co-eluted pri-let-7f1 stained with SybrGold.

Supplement Tables:

Table-S1- The data collection and model statistics for different MP^{he}-pri-let-7 cryo-EM structures.

Table-S2- The sequences for different RNA substrates used in the study. The mutated nucleotides are underlined.

Figure S1-

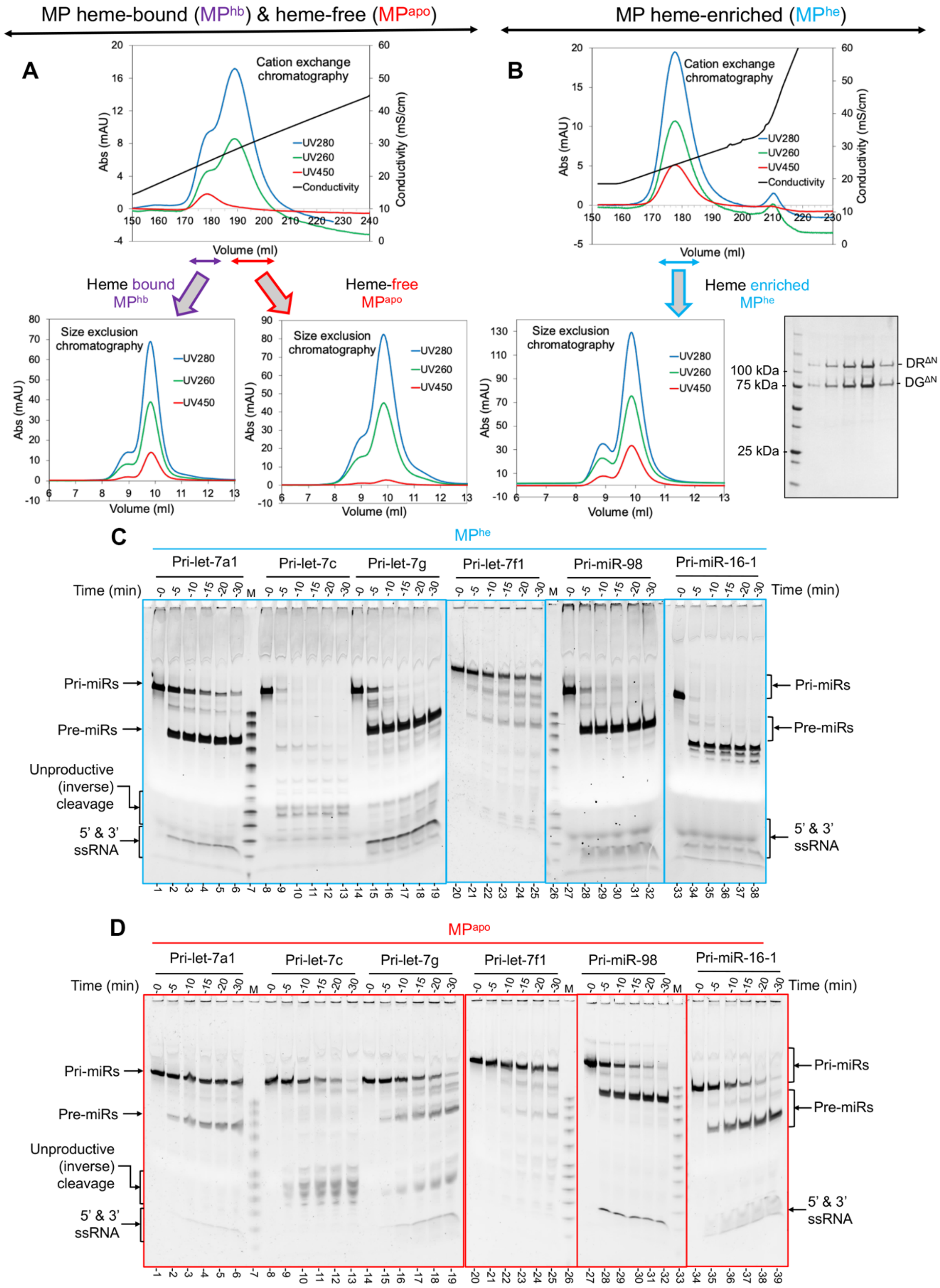

MP heme-bound (MPhb) & heme-free (MPapo)
MP heme-enriched (MPhe)
A
Cation exchange chromatography
Abs (mAU)
Conductivity (mS/cm)
UV280
UV260
UV450
Conductivity
Volume (ml)
B
Heme bound MPhb
Heme-free MPapo
Heme enriched MPhe
Size exclusion chromatography
100 kDa
75 kDa
25 kDa
DRΔN
DGΔN
C
MPhe
Pri-let-7a1
Pri-let-7c
Pri-let-7g
Pri-let-7f1
Pri-miR-98
Pri-miR-16-1
Time (min)
Pri-miRs
Pre-miRs
Unproductive (inverse) cleavage
5' & 3' ssRNA
D
MPapo

Figure S2-

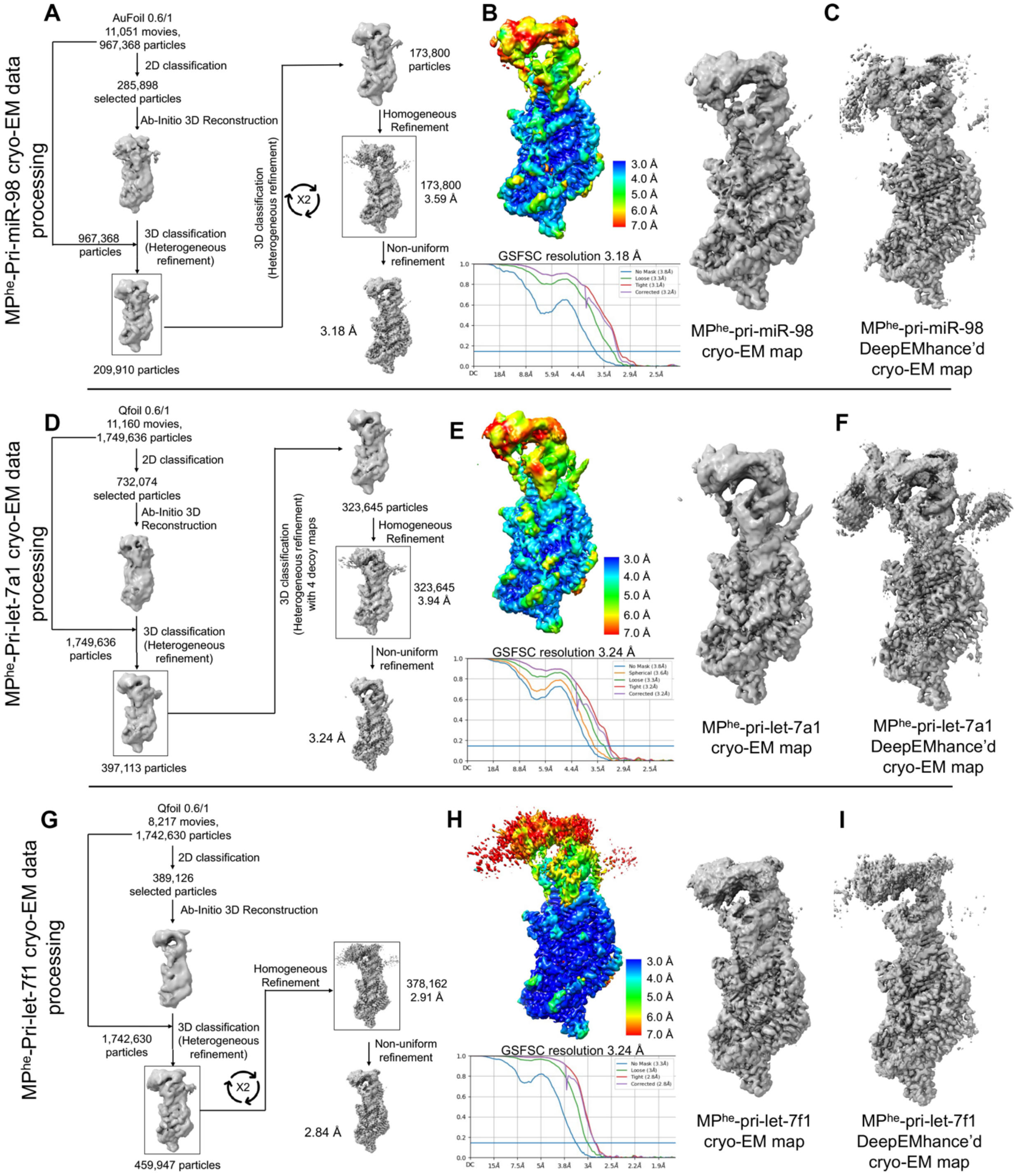
A
AuFoil 0.6/1
11,051 movies,
967,368 particles
2D classification
285,898
selected particles
Ab-Initio 3D Reconstruction
967,368
particles
3D classification
(Heterogeneous
refinement)
209,910 particles
3D classification
(Heterogeneous refinement)
X2
173,800
particles
Homogeneous
Refinement
173,800
3.59 Å
Non-uniform
refinement
3.18 Å
MPhe-Pri-miR-98 cryo-EM data processing
B
3.0 Å
4.0 Å
5.0 Å
6.0 Å
7.0 Å
GSFSC resolution 3.18 Å
C
MPhe-pri-miR-98
cryo-EM map
MPhe-pri-miR-98
DeepEMhance'd
cryo-EM map
D
Qfoil 0.6/1
11,160 movies,
1,749,636 particles
2D classification
732,074
selected particles
Ab-Initio 3D
Reconstruction
1,749,636
particles
3D classification
(Heterogeneous
refinement)
397,113 particles
3D classification
(Heterogeneous refinement)
with 4 decoy maps
323,645 particles
Homogeneous
Refinement
323,645
3.94 Å
Non-uniform
refinement
3.24 Å
MPhe-Pri-let-7a1 cryo-EM data processing
E
3.0 Å
4.0 Å
5.0 Å
6.0 Å
7.0 Å
GSFSC resolution 3.24 Å
F
MPhe-pri-let-7a1
cryo-EM map
MPhe-pri-let-7a1
DeepEMhance'd
cryo-EM map
G
Qfoil 0.6/1
8,217 movies,
1,742,630 particles
2D classification
389,126
selected particles
Ab-Initio 3D Reconstruction
1,742,630
particles
3D classification
(Heterogeneous
refinement)
459,947 particles
X2
Homogeneous
Refinement
378,162
2.91 Å
Non-uniform
refinement
2.84 Å
MPhe-Pri-let-7f1 cryo-EM data processing
H
3.0 Å
4.0 Å
5.0 Å
6.0 Å
7.0 Å
GSFSC resolution 3.24 Å
I
MPhe-pri-let-7f1
cryo-EM map
MPhe-pri-let-7f1
DeepEMhance'd
cryo-EM map

Figure S3-

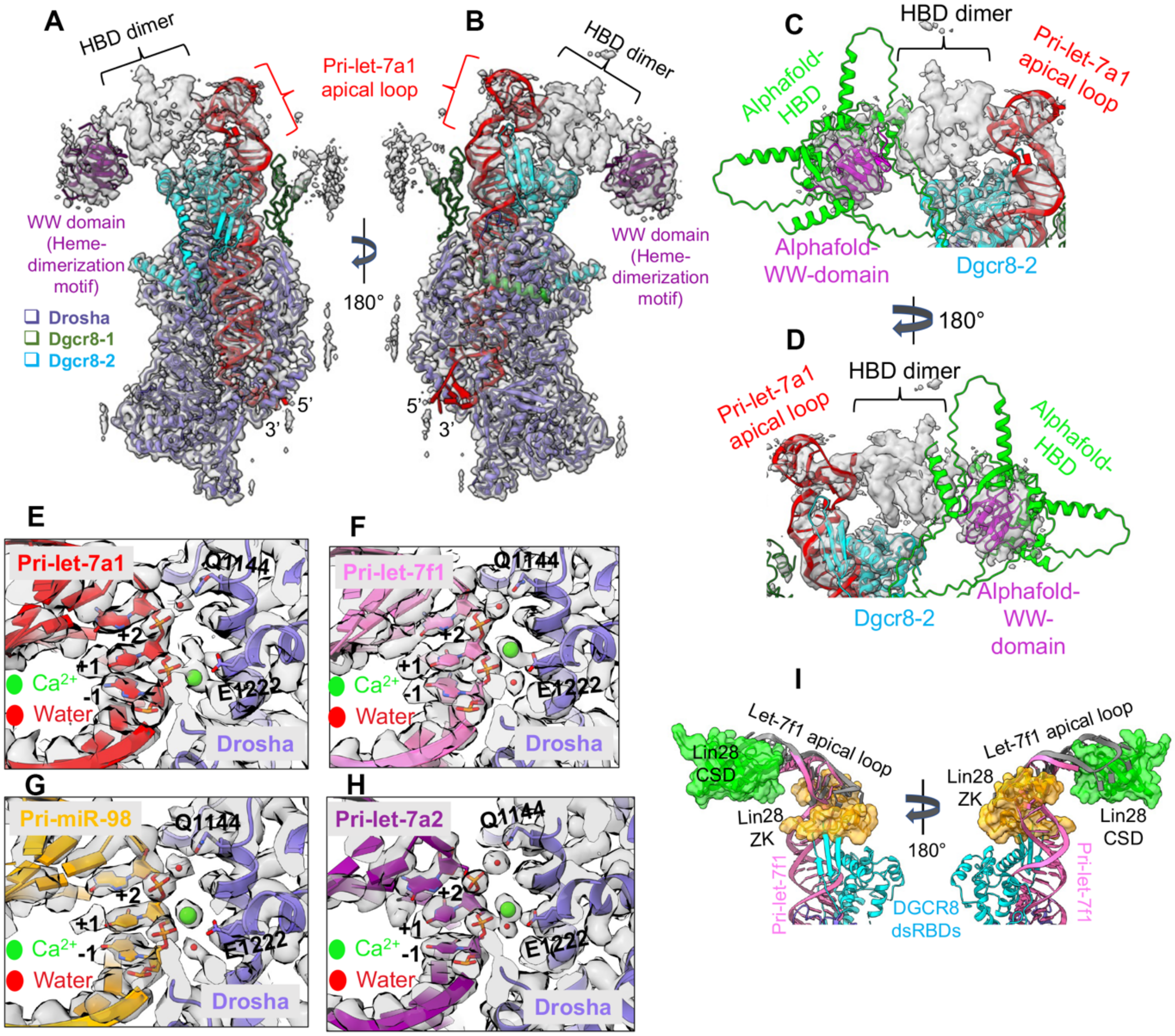

A
HBD dimer
Pri-let-7a1
apical loop
WW domain
(Heme-
dimerization
motif)
Drosha
Dgcr8-1
Dgcr8-2
180°
5'
3'
B
HBD dimer
WW domain
(Heme-
dimerization
motif)
5'
3'
C
HBD dimer
Pri-let-7a1
apical loop
Alphafold-
HBD
Alphafold-
WW-domain
Dgcr8-2
180°
D
HBD dimer
Pri-let-7a1
apical loop
Alphafold-
HBD
Dgcr8-2
Alphafold-
WW-
domain
E
Pri-let-7a1
Q1144
+2
+1
-1
Ca2+
Water
E1222
Drosha
F
Pri-let-7f1
Q1144
+2
+1
-1
Ca2+
Water
E1222
Drosha
G
Pri-miR-98
Q1144
+2
+1
-1
Ca2+
Water
E1222
Drosha
H
Pri-let-7a2
Q1144
+2
+1
-1
Ca2+
Water
E1222
Drosha
I
Let-7f1 apical loop
Lin28
CSD
Lin28
ZK
Pri-let-7f1
DGCR8
dsRBDs
180°
Let-7f1 apical loop
Lin28
ZK
Lin28
CSD
Pri-let-7f1

Figure S4-

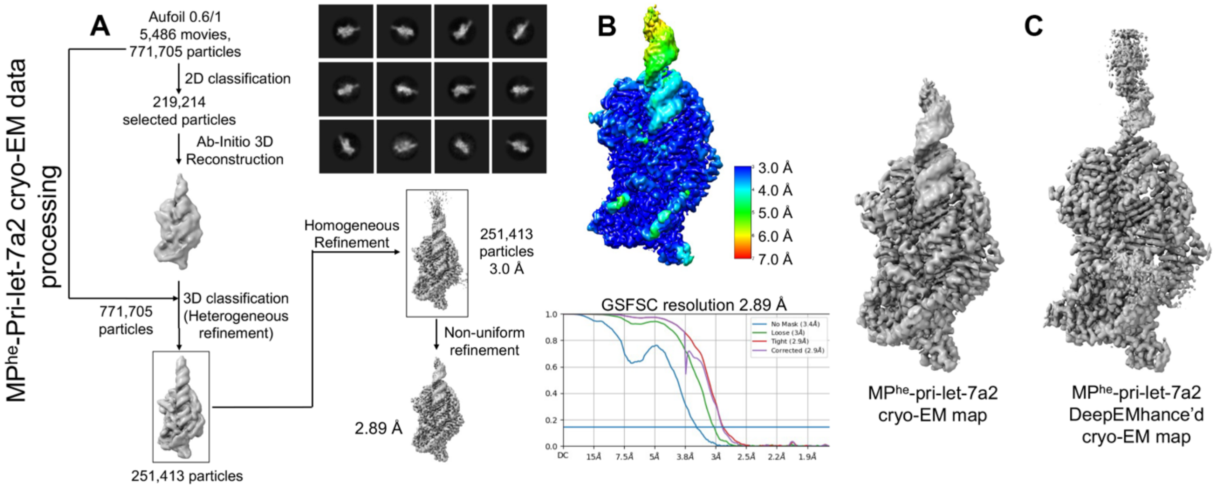

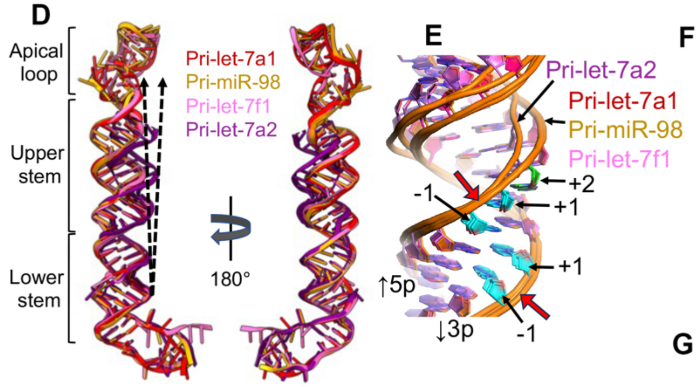

F

Base-pair	Shear (Å)							
	Pri-let-7a1		Pri-let-7f1		Pri-miR-98		Pri-let-7a2	
	G-C	-0.20	G-C	-0.20	G-U	-1.67	A-U	0.04
	A-U	0.30	A-U	0.04	A-U	0.11	G-U	-1.84
**5' catalytic pair**	**G-U**	**-2.1**	**G-U**	**-2.70**	**G-U**	**-2.76**	**U-U**	**-1.10**
	A-U	0.11	G-C	-0.17	G-C	-0.15	U-C	0.57
3' catalytic pair	G-C	-0.20	A-C	-2.20	G-C	-0.17	G-C	0.23
	G-C	-0.16	G-C	-0.16	G-C	-0.13	G-U	-1.13

Base-pair parameters

G

Steps	Rise (Å)							
	Pri-let-7a1		Pri-let-7f1		Pri-miR-98		Pri-let-7a2	
	AG/CU	3.21	AG/CU	3.36	AG/UU	3.10	GA/UU	3.83
	GA/UU	3.30	GA/UU	3.50	GA/UU	3.21	UG/UU	3.12
**5' catalytic step**	**AG/UU**	**4.82**	**GG/UC**	**4.84**	**GG/UC**	**4.80**	**UU/UC**	**3.30**
	GA/UC	3.45	AG/CC	3.40	GG/CC	3.52	GU/CC	3.40
3' catalytic step	GG/CC	3.44	GA/CC	3.28	GG/CC	3.34	GG/CU	3.52

Base-pair step parameters

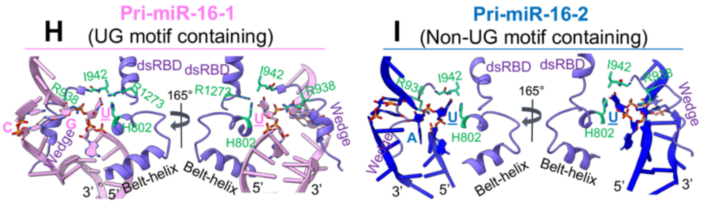

Figure S5-

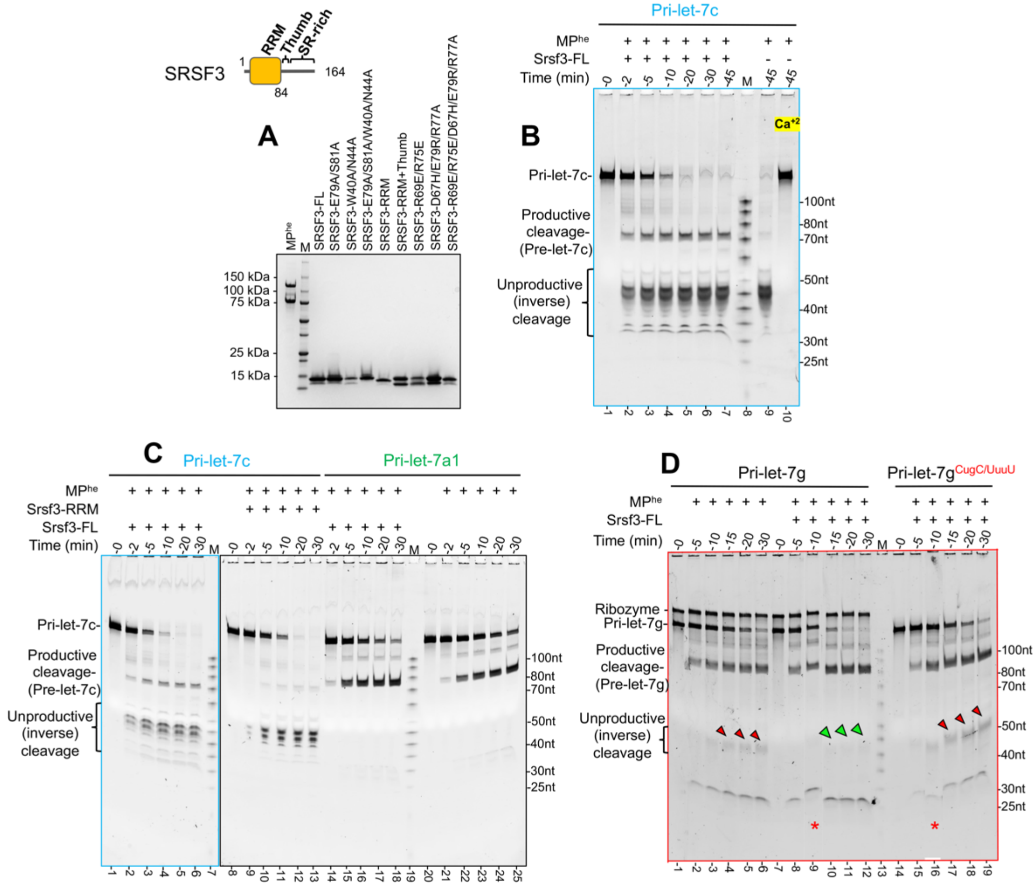
SRSF3
RRM
Thumb
SR-rich
1
84
164
A
MP^he
M
SRSF3-FL
SRSF3-E79A/S81A
SRSF3-W40A/N44A
SRSF3-E79A/S81A/W40A/N44A
SRSF3-RRM
SRSF3-RRM+Thumb
SRSF3-R69E/R75E
SRSF3-D67H/E79R/R77A
SRSF3-R69E/R75E/D67H/E79R/R77A
150 kDa
100 kDa
75 kDa
25 kDa
15 kDa
B
Pri-let-7c
MP^he
Srsf3-FL
Time (min)
Ca+2
Pri-let-7c-
Productive cleavage- (Pre-let-7c)
Unproductive (inverse) cleavage
100nt
80nt
70nt
50nt
40nt
30nt
25nt
C
Pri-let-7c
Pri-let-7a1
MP^he
Srsf3-RRM
Srsf3-FL
Time (min)
Pri-let-7c-
Productive cleavage- (Pre-let-7c)
Unproductive (inverse) cleavage
D
Pri-let-7g
Pri-let-7g^CugC/UuuU
MP^he
Srsf3-FL
Time (min)
Ribozyme
Pri-let-7g
Productive cleavage- (Pre-let-7g)
Unproductive (inverse) cleavage

Figure S6-

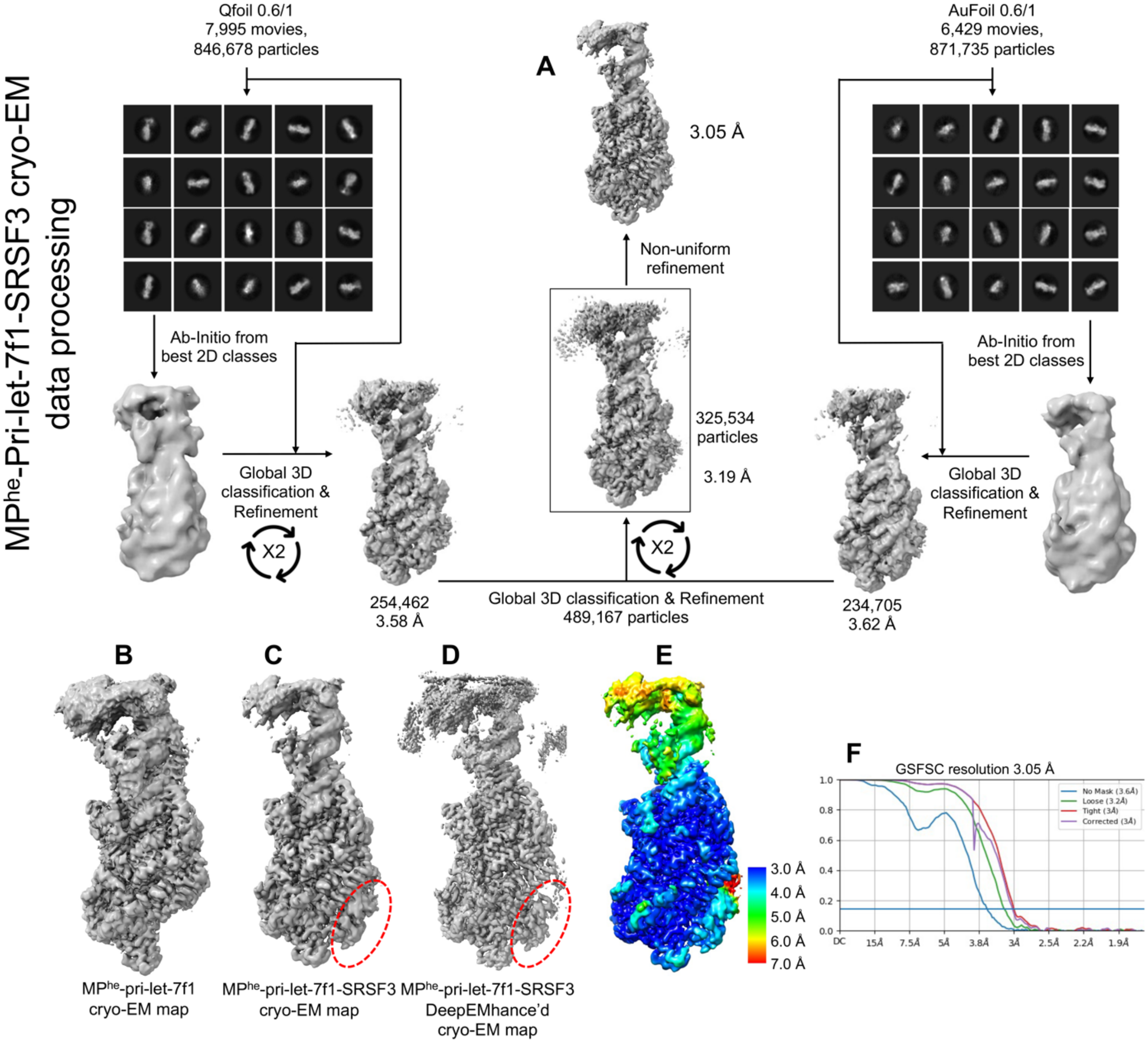
MP^{he}-Pri-let-7f1-SRSF3 cryo-EM data processing
A
Qfoil 0.6/1
7,995 movies,
846,678 particles
AuFoil 0.6/1
6,429 movies,
871,735 particles
3.05 Å
Non-uniform refinement
Ab-Initio from best 2D classes
Ab-Initio from best 2D classes
325,534 particles
3.19 Å
Global 3D classification & Refinement
Global 3D classification & Refinement
X2
X2
254,462
3.58 Å
Global 3D classification & Refinement
489,167 particles
234,705
3.62 Å
B
C
D
E
MP^{he}-pri-let-7f1 cryo-EM map
MP^{he}-pri-let-7f1-SRSF3 cryo-EM map
MP^{he}-pri-let-7f1-SRSF3 DeepEMhance'd cryo-EM map
3.0 Å
4.0 Å
5.0 Å
6.0 Å
7.0 Å
F
GSFSC resolution 3.05 Å
No Mask (3.6Å)
Loose (3.2Å)
Tight (3Å)
Corrected (3Å)
1.0
0.8
0.6
0.4
0.2
0.0
DC
15Å
7.5Å
5Å
3.8Å
3Å
2.5Å
2.2Å
1.9Å

Figure S7-

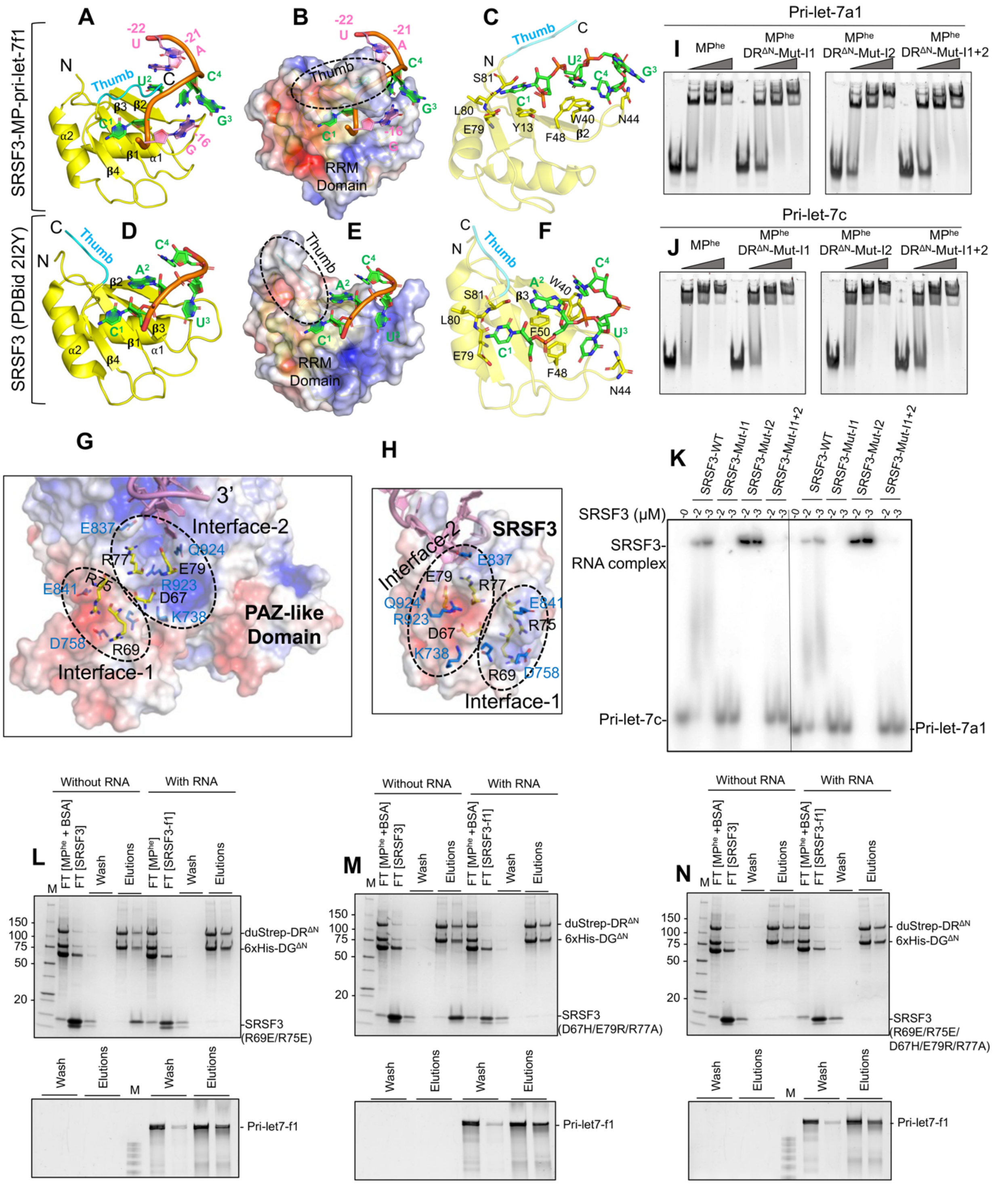

A
B
C
D
E
F
G
H
I
J
K
L
M
N
SRSF3-MP-pri-let-7f1
SRSF3 (PDBid 2I2Y)
Thumb
RRM Domain
Interface-1
Interface-2
PAZ-like Domain
SRSF3
Pri-let-7a1
Pri-let-7c
MPhe
MPhe DRΔN-Mut-I1
MPhe DRΔN-Mut-I2
MPhe DRΔN-Mut-I1+2
SRSF3-WT
SRSF3-Mut-I1
SRSF3-Mut-I2
SRSF3-Mut-I1+2
SRSF3 (μM)
SRSF3-RNA complex
Without RNA
With RNA
FT [MPhe + BSA]
FT [SRSF3]
Wash
Elutions
FT [MPhe]
FT [SRSF3-f1]
duStrep-DRΔN
6xHis-DGΔN
SRSF3 (R69E/R75E)
SRSF3 (D67H/E79R/R77A)
SRSF3 (R69E/R75E/D67H/E79R/R77A)
Pri-let7-f1

Table-S1: data collection and refinement statistics for cryo-EM structure of MP^{he}-pri-let-7s.

Cryo-EM Map	MP^{he}-pri-let-7a1	MP^{he}-pri-miR-98	MP^{he}-pri-let-7f1	MP^{he}-pri-let-7a2	MP^{he}-pri-let-7f1-SRSF3
**Data collection and processing**					
Microscope	Titan Krios				
Voltage (kV)	300				
Detector	K3				
Magnification	81,000x	81,000x	105,000x	105,000x	105,000x
Pixel size (Å)	1.1	1.1	0.856	0.856	0.856
Data collection software	EPU	EPU	EPU	EPU	EPU
Defocus range (μm)	0.6 – 2.2	0.7 – 2.2	0.6 – 2.2	0.6 – 2.0	0.7 – 2.2
Total exposure (e^{-}/Å^2)	72.16	42.6	77.6	78	76.6
Frames/exposure	30	30	30	30	30
Exposure/frame (e^{-}/Å^2)	2.405	1.42	2.59	2.6	2.55
Micrographs collected	11,160	11,051	8,217	5,486	7,995 (Q-foil)<br>6,429 (Au-foil)
Total extracted particles	1,749,636	967,368	1,742,639	771,705	846,678 (Q-foil)<br>871,735 (Au-foil)
Particles for 3D reconstruction	397,113	209,910	459,947	251,413	489,167
Final particles	323,645	173,800	378,162	251,413	325,534
Symmetry	C1	C1	C1	C1	C1
**Map resolution (unmasked/masked)**					
GSFSC 0.143	3.8/3.2	3.8/3.2	3.3/2.8	3.4/2.9	3.6/3.0
**Refinement and model validation**					
**Model resolution (unmasked/masked)**					
FSC 0.143	3.3/3.2	3.2/3.2	3.1/3.0	3.1/3.0	3.2/3.2
FSC 0.5	4.5/4.4	4.5/4.4	4.3/4.2	4.2/4.2	4.6/4.6
Map CC mask/volume	0.68/0.71	0.70/0.72	0.63/0.65	0.65/0.67	0.60/0.63
Protein residue	1256	1184	1180	966	1257
Nucleic acid	96	97	95	72	100
Water	1	2	2	2	2
**B-factors (Å^2)**					
Protein	180.90	178.97	178.25	154.41	167.82
Nucleotide	76.85	81.97	80.11	83.79	78.78
**RMS Deviations**					
Bond length (Å)	0.004	0.005	0.003	0.002	0.003
Bond angle (°)	0.582	0.577	0.489	0.490	0.465
**Ramachandran statistics**					
Favored (%)	94.86	90.63	97.17	98.23	97.34
Allowed (%)	5.16	9.37	2.83	2.77	2.66
Outlier (%)	0.0	0.0	0.0	0.0	0.0
Rotamer outliers (%)	0.09	0.0	1.04	0.81	0.09
CaBLAM outliers (%)	2.1	1.5	2.3	1.5	2.1
Clash score (all atoms)	10.51	6.14	3.96	3.65	4.84
MolProbity score	1.90	1.57	1.34	1.15	1.37
FSC is Fourier shell correlation, and RMSD is root-mean-square deviation.					

Table S2. Pri-miRNA sequences used in this study, related to STAR Methods.
Mutations of wild type (WT) sequences are underlined.

**RNA name**	**Sequence**
Pri-let-7a1:	UGGAUGUUCUCUUCACUGUGGGAUGAGGUAGUAGGUUGUAU<br>AGUUUUAGGGUCACACCCACCACUGGGAGAUAACUAUACAAU<br>CUACUGUCUUUCCUAACGUGAUAGAAAAGUCUGCAU
Pri-let-7a2:	UCCAGCCAUUGUGACUGCAUGCUCCCAGGUUGAGGUAGUAG<br>GUUGUAUAGUUUAGAAUUACAUCAAGGGAGAUAACUGUACAG<br>CCUCCUAGCUUUCCUUGGGUCUUGCACUAAACAACAUGGUG<br>AGA
Pri-let-7c:	ACAUUGGAAGCUGUGUGCAUCCGGGUUGAGGUAGUAGGUUG<br>UAUGGUUUAGAGUUACACCCUGGGAGUUAACUGUACAACCU<br>UCUAGCUUUCCUUGGAGCACACUUGAGCCGUCGAGGAAUUC<br>UU
Pri-let-7f1:	AUUCCAGAAGAAAACAUUGCUCUAUCAGAGUGAGGUAGUAGA<br>UUGUAUAGUUGUGGGGUAGUGAUUUUACCCUGUUCAGGAGA<br>UAACUAUACAAUCUAUUGCCUUCCCUGAGGAGUAGACUUGCU<br>GCAUUAUUUUCU
Pri-let-7g:	UUCCUUUUGCCUGAUUCCAGGCUGAGGUAGUAGUUUGUACA<br>GUUUGAGGGUCUAUGAUACCACCCGGUACAGGAGAUAACUG<br>UACAGGCCACUGCCUUGCCAGGAACAGCGCGCCAGCUGCCA<br>AG
Pri-miR-98:	AGGAUUCUGCUCAUGCCAGGGUGAGGUAGUAAGUUGUAUUG<br>UUGUGGGGUAGGGAUAUUAGGCCCCAAUUAGAAGAUAACUA<br>UACAACUUACUACUUUCCCUGGUGUGUGGCAUAUUCAGCAU
Pri-let-7c$^{CNNC/UUUU}$:	ACAUUGGAAGCUGUGUGCAUCCGGGUUGAGGUAGUAGGUUG<br>UAUGGUUUAGAGUUACACCCUGGGAGUUAACUGUACAACCU<br>UCUAGCUUUCCUUGGAGCACACUUGAGC**UUUU**GAGGAAUUC<br>UU
Pri-let-7g$^{CNNC/UUUU}$:	UUCCUUUUGCCUGAUUCCAGGCUGAGGUAGUAGUUUGUACA<br>GUUUGAGGGUCUAUGAUACCACCCGGUACAGGAGAUAACUG<br>UACAGGCCACUGCCUUGCCAGGAACAGCGCGCCAG**UUUU**CA<br>AG
Pri-miR-16-1:	AUAGCAAUGUCAGCAGUGCCUUAGCAGCACGUAAAUAUUGGC<br>GUUAAGAUUCUAAAAUUAUCUCCAGUAUUAACUGUGCUGCUG<br>AAGUAAGGUUGACCAUACUCUA
Pri-miR-16-2:	CUGACAUACUUGUUCCACUCUAGCAGCACGUAAAUAUUGGCG<br>UAGUGAAAUAUAUAUUAAACACCAAUAUUACUGUGCUGCUUU<br>AGUGUGACAGGGAUACAGCAA
